# Analysis of *CcGASA* family members in *Citrus clementina* (Hort. ex Tan.) by a genome-wide approach

**DOI:** 10.1186/s12870-021-03326-6

**Published:** 2021-12-01

**Authors:** Tianli Wu, Yun Zhong, Min Chen, Bo Wu, Ting Wang, Bo Jiang, Guangyan Zhong

**Affiliations:** 1 College of Horticulture and Landscape Architecture, Southwest University, Chongqing, 400716 China; 2 Institute of Fruit Tree Research, Guangdong Academy of Agricultural Sciences, Guangzhou, 510640 China; 3 Key Laboratory of South Subtropical Fruit Biology and Genetic Resource Utilization, Ministry of Agriculture, Guangzhou, 510640 China; 4 Key Laboratory of Tropical and Subtropical of Fruit Tree Research, Science and Technology Department of Guangdong Province, Guangzhou, 510640 China

**Keywords:** GASA, Citrus, Phylogenetic tree, Promoter, Transcription factor, Protein interaction

## Abstract

The Gibberellic Acid Stimulated Arabidopsis (GASA) proteins were investigated in the study to help understand their possible roles in fruit trees, particularly in *Citrus*. A total of 18 CcGASA proteins were identified and characterized in *Citrus clementina* via a genome-wide approach. It was shown that the CcGASA proteins structurally shared a conserved GASA domain but varied considerably in primary sequences and motif compositions. Thus, they could be classified into three major phylogenetic groups, G1~G3, and two groups, G1 and G3 could be further classified into subgroups. The cis- elements on all *CcGASA* promoters were identified and categorized, and the associated transcription factors were predicted. In addition, the possible interactions between the CcGASA proteins and other proteins were predicted. All the clues suggested that these genes should be involved in defense against biotic and abiotic stresses and in growth and development. The notion was further supported by gene expression analysis that showed these genes were more or less responsive to the treatments of plant hormones (GA_3_, SA, ABA and IAA), and infections of citrus canker pathogen *Xanthomonas citri.* It was noted that both the segmental and the tandem duplications had played a role in the expansion of the *CcGASA* gene family in *Citrus.* Our results showed that the members of the *CcGASA* gene family should have structurally and functionally diverged to different degrees, and hence, the representative group members should be individually investigated to dissect their specific roles.

**Supplementary Information:**

The online version contains supplementary material available at 10.1186/s12870-021-03326-6.

## Introduction

The Snakin/Gibberellic Acid Stimulated in Arabidopsis (GASA) is a unique multigene family. Since the isolation of GAST1 (Gibberellic Acid Stimulated Transcript 1) from tomato [43], many GASA protein family members have been characterized in different species, such as potato [42], common wheat [13], soybean [2], *Arabidopsis* [6, 38], petunia [8], rice [15], apple [14], grapevine [1] and maize [56]. A comprehensive genome sequence analysis of 33 plant species revealed approximately 445 Snakin/GASA protein encoding genes [45]. Further bioinformatics data mining showed that the *Snakin/GASA* genes were present in all well-characterized or sequenced plant species but were completely absent in moss and green algae [17]. It is known that the GASA family peptides share a conserved C-terminal domain, designated as GASA. The GASA domain contains 12 cysteine residues (Cys-motif) arranged in the pattern of “XnCX_3_CX_3_CX_8(9)_CX_3_CX_2_CCX_2_CX_1(3)_C_11_CPC_11(14)_KCP” (where: X represents any of the 20 non-cysteine amino acids; P and K represent proline and lysine, respectively) [48]. A typical GASA protein also possesses a putative signal peptide at the N-terminus and a variable region in the middle of the sequence.

GASA proteins are known to play diverse roles in plants. They are involved in the regulation of growth and development processes, including cell division [34], stem elongation [8], floral induction [36], seed germination [38, 39], lateral root formation [56] and fruit development [33]. The GASA proteins are also linked to stress responses, such as resistance to heat [26], drought, and paclobutrazol (PBZ) stresses [51], tolerance to salt and oxidative stresses [3], and modulation of reactive oxygen species (ROS) [47]. Moreover, GASA proteins have shown suppressive effects on a wide range of bacterial and fungal pathogens. Purified StSN1 peptide, for example, was found in an in *vitro* challenge experiment to be toxic to several fungal pathogens like *Fusarium solani*, *Fusarium culmorum*, *Bipolaris maydis* and *Botrytis cinerea*, and to bacterial pathogens such as *Clavibacter michiganensis* subsp. *Sepedonicus* [46]. The GASA family proteins even exhibit anti-viral and anti-nematode activities, as exemplified by GmSN1 that enhances soybean mosaic virus resistance in both *Arabidopsis* and soybean [19] and by CaSn that promotes nematode-resistance in pepper [31]. The citrus CcGASA4 was shown to be highly induced in citrus leaves following infection of *Citrus tristeza virus* [54].

GASA family genes are implicated in the responses of plants to hormones such as gibberellin (GA), abscisic acid (ABA), salicylic acid (SA), jasmonic acid (JA) and ethylene (ETH). Most *GASA* genes including *Arabidopsis AtGASA4*, *6*, *7*, *8* and *13* [39, 57], rice *OsGASR1* and *2* [15], petunia *GIP 1*, *2*, *4* and *5* [8], maize *ZmGSL1*, *2*, *4*, *6* and *9* [56], were induced by exogenous GA treatment. However, some other GASA genes, such *as Arabidopsis AtGASA1*, *5*, *9* and *11* [57], and potato *StSN2* [9] were repressed by GA. Interestingly, some *GASA* genes exhibited tissue-specific responses to GA applications. *GsGASA1* expression was induced in leaves but repressed in roots by exogenous application of GA in *Glycine soja* [30]. *AtGASA4* was up-regulated in most, if not all, meristematic regions, presumably in actively dividing cells, but was down-regulated in cotyledons and leaves following GA treatment [6]. ABA was shown to induce the expression of *AtGASA2*, *3*, *5* and *14*, and inhibit the expression of *AtGASA7* and *9* in *Arabidopsis* [57]. The expression of *StSN2* was induced [9] whereas *Snakin-3* was down regulated by ABA treatment in potato [35]. In addition, some members of the *GASA* family, such as *GAST1* [37, 43], *StSN2* [9] and *GASA5* [57], were regulated by GA and ABA antagonism. The GA mediated increase in *GAST1* transcripts was partially inhibited by ABA in tomato [43]. *AtGASA1* was up-regulated by GA and down-regulated by ABA in *Arabidopsis* [37]. *StSN2* was up- and down- regulated by ABA and GA, respectively, in potato [9]. The expression of *GASA5* was repressed by GA_3_ and enhanced by ABA [57]. *GASA* genes are also responsive to other hormones. Brassinoesteroid (BR) synthesis was activated by OsGSR1 by directly regulating a BR biosynthetic enzyme [52]. The transcription of *HbGASA7-1*, *14* and *16* was significantly increased after ETH, SA, or JA treatment in *Hevea brasiliensis* [4].

Less information about the GASA genes is available for trees, particularly for fruit trees. Nevertheless, 14 *VvGASA* genes in grapevine (*Vitis vinifera* L.) [1], 26 *MdGASA* genes in apple (*Malus domestica*) [14] and a *CcGASA4* gene in citrus [54] were reported. Citrus is one of the most important fruit trees worldwide. A steady increase in global per capita consumption of citrus fruits has been witnessed in the past 30 years [32]. Citrus production is, however, being threatened by numerous biotic and abiotic stresses. Identification and functional analysis of citrus defense- and stress-related genes should deepen our understanding of the molecular mechanisms of stress-responses and lend helps in improving stress tolerance in plants. Considering that a citrus GASA gene was highly responsive to the *Citrus tristiza virus*, we performed a detailed bioinformatics analysis of the relevant gene family, aiming to provide first tier information for dissecting their exact roles in the defense of citrus against stresses.

## Materials and methods

### Identification of putative *Citrus clementina GASA* genes

The GASA protein sequences of *Arabidopsis*, apple [14] and grape [1] were downloaded from the EnsemblPlants online database (http://plants.ensembl.org/info/data/ftp/index.html) and analyzed using the HMMER (https://www.ebi.ac.uk/Tools/hmmer/) to build a model based on the GASA domain (Accession: pfam02704). The model was used to query the entire *C. clementina* genome to obtain all putative clementine mandarin GASA proteins (Supplementary Table S1). The integrity of the GASA domain of every CcGASA protein sequence was verified using the Simple Modular Architecture Research Tool (SMART: http://smart.embl.de/) [24]. All non-redundant putative protein sequences with conserved GASA domain were reserved and used for further analysis. GASA proteins from other three citrus species, pommelo (*Citrus maxima*)*,* sweet orange (*Citrus sinensis*) and trifoliate orange (*Poncirus trifoliata*), were also identified following the same protocol.

All CcGASA protein coding sequences, genomic sequences and the associated information such as accession number and chromosomic position, were downloaded from the Phytozome database (https://phytozome.jgi.doe.gov/pz/portal.html#). In addition, the physical location of each *CcGASA* gene on the genome was mapped by the Mapchart software.

### Analysis of physicochemical properties of the citrus GASA proteins

The physicochemical parameters of the CcGASA proteins were calculated by using the ProtParam (http://web.expasy.org/protparam) [16]. Protein putative subcellular locations and tertiary structures were predicted by using the WOLF PSORT II program (https://www.genscript.com/wolf-psort.html) [22] and the PHYRE2 engine (http://www.sbg.bio.ic.ac.uk/phyre2/html/page.cgi?id=index), respectively. The transmembrane helices were predicted using the TMHMM server v2.0 (http://www.cbs.dtu.dk/services/TMHMM-2.0/).

### Analysis for protein phylogenetic relationships and gene structures

A phylogenetic tree was generated based on 111 GASA protein sequences from different plant species including *C. clementina* (15), *C. sinensis* (14), *C. maxima* (13), *P. trifoliata* (14), *Arabidopsis thaliana* (15), *M. domestica* (26) and *V. vinifera* (14). The GASA sequences of *C. sinensis*, *C. maxima* and *P. trifoliata* were downloaded from the Orange (*C. sinensis*) Genome Annotation Project website (http://citrus.hzau.edu.cn/orange/). MEGA 7.0 software [28] was used to construct the phylogenetic tree by using the neighbor-joining (NJ) method. The parameters of the NJ method were as follows: 1000 bootstrap replications, “*p*-distance” model, “Uniform rates”, “partial deletion”, and 95% site coverage cutoff. The conserved regions within the CcGASA proteins were identified by using the MEME v5.2.0 (http://meme-suite.org/tools/meme) [7]. The *GASA* CDSs and their corresponding genomic sequences of the four *Citrus* species were compared to reveal exons and introns using the Gene Structure Display Server 2.0 (GSDS2.0, http://gsds.gao-lab.org/).

### Promoter analysis of *CcGASA* genes

Around 1.9-kb long promoter sequence upstream of the start codon (ATG) of each *CcGASA* gene was downloaded from the Phytozome12.0 database [18]. The cis-regulatory elements on the promoters were analyzed by using the Plant Cis-Acting Regulatory DNA Elements (PlantCARE) program (http://bioinformatics.psb.ugent.be/webtools/plantcare/html/) [29].

### Expression analysis of *CcGASA* genes

The 4-year-old *C. clementina* trees had been planted in plastic pots and used as experimental materials. For the biotic stress treatment, mature leaves of similar size and shape were picked and brought to lab in a humidified chamber, and immediately pierced at ten evenly spaced points at the back of each leaf with a syringe needle. The pierced leaves were placed face down in a try lined at the bottom with water-soaked filter papers. Ten micro litter of *Xanthomonas citri* subsp. *Citri* (Xcc) cell cultures, diluted to 5 × 10^8^ cfu/mL (OD_600_ ≈ 0.3), was applied to cover the pierced holes. For controls, 10 μL of water was applied. The trays were then misted with water and covered with plastic films, and sampled at 0 h, 12 h, 24 h, 48 h, and 72 h. Samples were immediately frozen in liquid nitrogen, and then stored in a -80°C refrigerator until use. For hormone treatment, trees were sprayed with 100 μΜ of GA_3_, 2 mΜ of SA, 100 μM of indole-3-acetic acid (IAA) and 200 μΜ of ABA, and leaves were harvested after 3h, 6h, 12h and 24h, flash frozen in liquid nitrogen, and stored at −80°C. Three biological replicates were used in the study.

Total RNA was extracted from frozen leaf samples using the Polysaccharide Polyphenol plant total RNA rapid extraction kit (Bioteke, China) following the manufacturer’s instructions. RNA concentrations were determined using the NanoDrop2000C (ThermoFisher Scientific, USA), and RNA quality was evaluated by ratios of OD_260_ / OD_280_. cDNA was synthesized from total RNA using the PrimeScript^TM^ RT reagent kit with gDNA Eraser (TaKaRa, Japan). The quantitative real-time PCR (qRT-PCR) experiment was performed on a thermo-cycler, QuantStudio5 (ABI, USA). Each tube of qRT-PCR solution contained 10.0 μL iTaq^TM^ Universal SYBR® Green Supermix (Bio-Rad, USA), 1.0 μL primer pair F/R, 2.0 μL cDNA, and 6.0 μL dH_2_O. The thermo-cycler was programed as follows: an initial incubation at 95°C for 10 min and followed by 40 cycles of 95°C for 5 s + 60°C for 20 s. For melting-curve analysis, the program was set to 95°C for 15 s, 60°C for 2 min and then the temperature was progressively increased to 95°C at a constant rate of 0.2°C/s. The primers used were shown in Supplementary Table S2. The *Actin* (GenBank accession: XM_006427792) was used as the reference gene. Since three proteins, CcGASA16, CcGASA17 and CcGASA18, were predicted to be derived from a single gene sequence (Ciclev10006243m.g) via alternative splicing, it was impossible to design primers to separate *CcGASA18* from *CcGASA16* and *CcGASA17*, and hence only *CcGASA16* and *CcGASA17* were eventually analyzed. For quality controls, three technical replicates were also used in addition to three biological replicates. The relative expression level, shown as the ratio of the analyzed gene to the reference gene, was determined by calculating the 2 ^−ΔΔCt^ (ΔCt = Ct *CcGASA* -Ct *Actin*). Data were statistically analyzed using ANOVA. Duncan’s LSD multiple range test (p ≤ 0.05) was performed to reveal significant changes. Figures were drawn by using the Origin 2019b.

The leaves, stems, young fruits and roots from healthy *C. clementina* trees were subjected to transcriptome profiling in a commercial biotech company, Oebiotech Company (China; https://www.oebiotech.com/) to reveal possible tissue-specific expression patterns. Briefly, total RNA was isolated from samples and used to prepare RNA-seq libraries. The libraries were then sequenced on Illumina Genome Analyzer platform. Clean reads were obtained by passing the row sequencing data through all quality control procedures, and were mapped to the *C. clementina* genome sequence using the HISAT2 software. The expression level of each gene in the RNA-seq libraries was calculated as the FPKM (Fragments Per Kilobase of transcript per Million fragments mapped) by using the Cuffquant and Cuffnorm software. The differentially expressed transcripts, defined by a fold change (|log2Fold Change|) of greater than 1 and a *P* value (false discovery rate, FDR) of less than 0.05, between samples were identified by using the DESeq2 [5] software.

### Synteny analysis and calculation of Ka/Ks ratio for duplicated genes

*CcGASA* gene duplication events were identified according to the criteria proposed by Holub [21]. The Basic Circos function of TBtools software was used to show the interspersed segmental duplications using the data from the PLAZA (https://bioinformatics.psb.ugent.be/plaza/versions/plaza_v4_5_dicots/) [49]. The Ka (non-synonymous substitution rate) and Ks (synonymous substitution rate) between the duplicated genes were calculated using the online tool PAL2NAL (http://www.bork.embl.de/pal2nal/index.cgi?example=Yes#RunP2N). Mode of selection acting on the duplicated genes was evaluated through Ka/Ks ratio, and a positive, negative or neutral selection was considered when the ratio was > 1, < 1, or = 1, respectively. The gene loci of GASAs were extracted from the annotation gff3-file on the EnsemblPlants and the Orange (*C. sinensis*) Genome Annotation Project online database. Collinear pairs were extracted using TBtools [12] to identify syntenic blocks and duplications within the *GASAs* across the whole genomes of 7 species, *C. clementina*, *C. sinensis*, *C. maxima*, *P. trifoliata*, *A. thaliana*, *M. domestica* and *V. vinifera.* The collinearity map between these species was drawn with the help of MCScan X (TBtools software) program.

### Analysis of transcription factor regulatory network and protein interaction network involving CcGASA proteins

Transcription factor (TFs) network prediction was performed online at the threshold parameter *p*-value ≤ 1e-5 on the Plant Transcriptional Regulatory Map (PTRM) website (http://plantregmap.gao-lab.org/regulation_prediction.php), using all the *CcGASA* sequences as an input. The Cytoscape 3.8 software was used to visualize the transcription factor regulatory network [27]. The predicted TFs were subjected to GO analyses on the Omicshare cloud platform (https://www.omicshare.com/tools/). The functional interacting network models of CcGASA proteins were predicted using the web program STRING 11.0 (http://string-db.org). The confidence parameter was set at a threshold of 0.40, and for other parameters the default values were used.

## Results

### Genome-wide distribution of *C. clementina* GASA genes and features of their deduced proteins

**Fig. 1:**
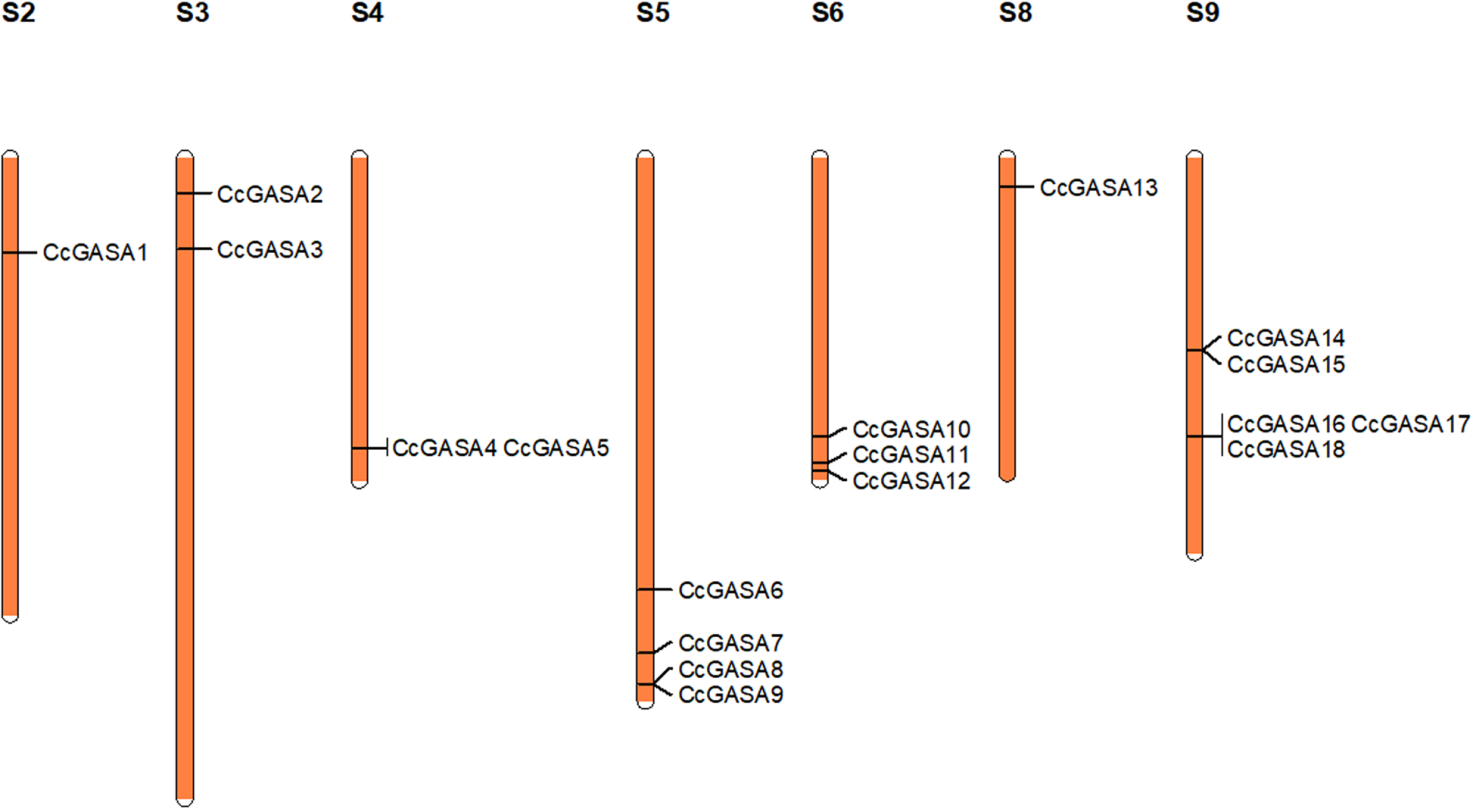
Chromosomal locations and duplications of citrus *CcGASA* genes. The scaffolds(S) number is indicated above each bar. The scaffold size is indicated by its relative length using the information from Phytozome

Eighteen putative CcGASA proteins were found by searching the *C. clementine* protein database against a model built from other known plant GASA proteins (Table 1), and correspondingly, 15 *CcGASA* genes were identified. The proteins were sequentially designated according to their chromosomal locations as CcGASA1–18 in this study (Fig. 1). Comparison between CcGASA4 and CcGASA5 showed that both are coded by the same gene, Ciclev10033115m.g. Similarly, CcGASA16, 17 and 18 are coded by Ciclev10006243m.g. It should be noted that Ciclev10013454m, previously designated as *CcGASA4* [54], was re-designated as CcGASA12 in this study. As shown in Fig. 1, the 15 *CcGASA* genes were located on 7 scaffolds on the *C. clementina* genome. More specifically, 4 of them were on scaffold 5, 3 on scaffold 6, 3 on scaffold 9, 2 on Scaffold 3, 1 on scaffold 2 and 1 on scaffold 4.

**Table 1:** Detailed information of citrus *CcGASA* genes

Transcript ID	Protein Name	Scaffold	Start Sit	End Sit	Strand	No. of Exons	CDS (bp)	Protein (A.A)
Ciclev10017244m	CcGASA1	S2	7477430	7479641	forward	4	342	113
Ciclev10022925m	CcGASA2	S3	2774465	2775592	forward	4	351	116
Ciclev10023012m	CcGASA3	S3	7235575	7236465	forward	3	312	103
Ciclev10033135m	CcGASA4	S4	23072592	23073840	reverse	3	342	107
Ciclev10033115m	CcGASA5	S4	23072592	23073840	reverse	3	342	113
Ciclev10002979m	CcGASA6	S5	34404147	34405161	reverse	2	267	88
Ciclev10002796m	CcGASA7	S5	39444681	39445917	forward	4	432	143
Ciclev10002927m	CcGASA8	S5	41913536	41914321	reverse	3	315	104
Ciclev10002984m	CcGASA9	S5	41920802	41921611	reverse	2	264	87
Ciclev10013200m	CcGASA10	S6	22210589	22211749	forward	3	288	95
Ciclev10012786m	CcGASA11	S6	24264177	24265636	reverse	4	621	206
Ciclev10013454m	CcGASA12	S6	24913375	24914177	forward	4	321	106
Ciclev10029695m	CcGASA13	S8	2308972	2309555	forward	2	267	88
Ciclev10006931m	CcGASA14	S9	15301341	15301778	reverse	2	213	70
Ciclev10006668m	CcGASA15	S9	15311772	15312184	reverse	2	213	70
Ciclev10006310m	CcGASA16	S9	22139872	22141728	forward	3	291	96
Ciclev10006347m	CcGASA17	S9	22140369	22141728	forward	4	246	81
Ciclev10006243m	CcGASA18	S9	22140369	22141728	forward	1	357	118

*CDS* coding sequence

**Fig. 2:**
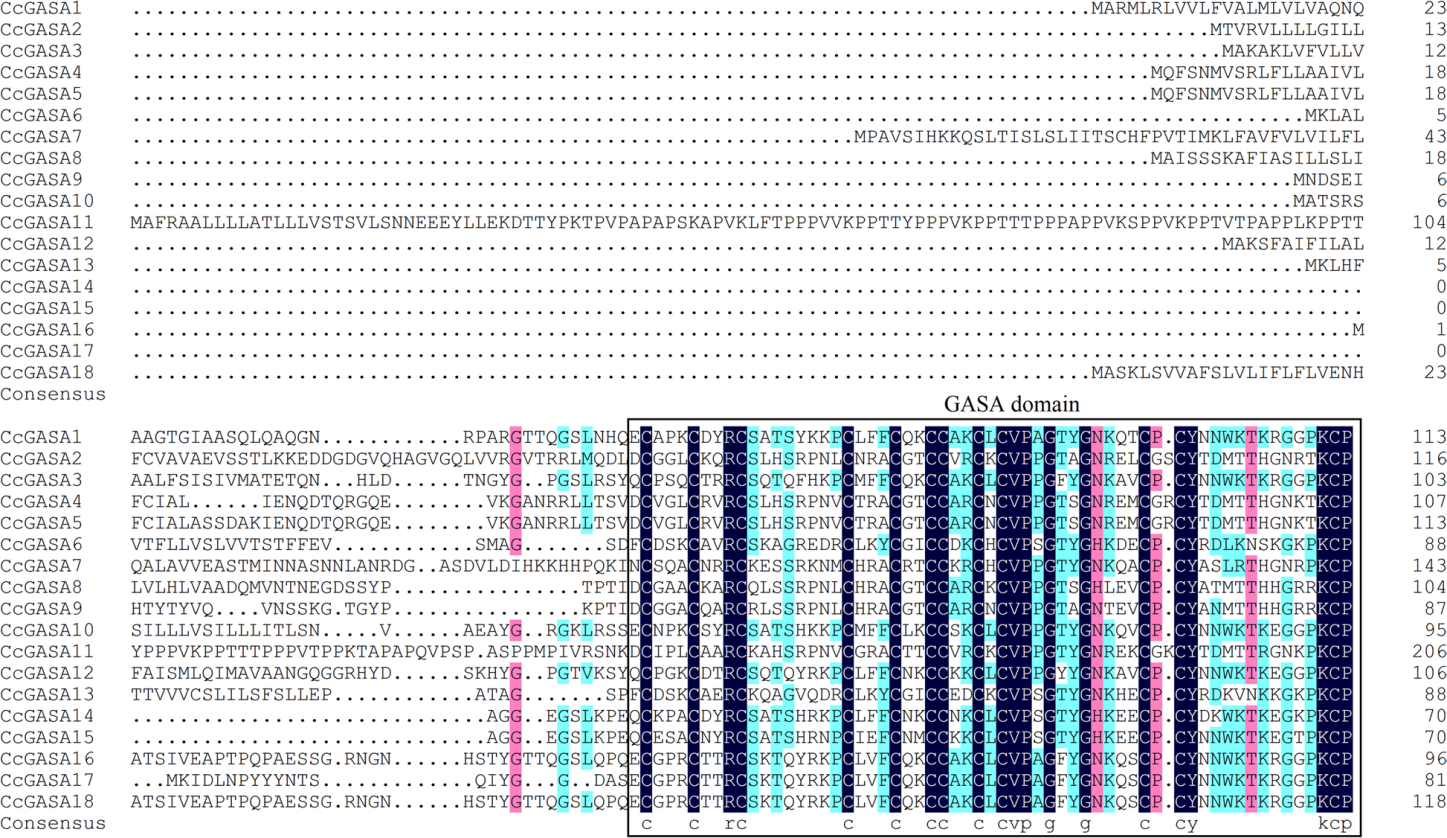
Amino acid sequence alignment of CcGASA proteins

The deduced protein sequences of the 18 CcGASA transcripts varied in length from 70 to 206 amino acids (Fig. 2). They were low molecular weight peptides, mostly less than 13kDa, although CcGASA7 (15.86 kDa) and CcGASA11 (21.78 kDa) were slightly larger (Table 2). All CcGASAs were relatively high in their isoelectric point (pI) values, for 7 of them had a pI value of higher than 8, and the others even higher than 9. They were mostly predicted to be unstable since 13 of them, except for CcGASA1, CcGASA2, CcGASA4, CcGASA5 and CcGASA6, had an instability index values of higher than 40. According to the Grand average of hydropathicity (GRAVY), the CcGASA proteins, excluding CcGASA2 and CcGASA8, were hydrophilic. Amino acid preference analysis showed that Cys, Lys, Ser, Leu and Pro were the preferable amino acids although Ala, Gly, Thr, Glu, Argand Tyr were also common. The aliphatic index values of the CcGASA proteins were different, varying from 25.14 to 84.74.

**Table 2:** Analysis of physicochemical properties of CcGASAs

Protein name	MW	PI	Major amino acid%	Instability index	Aliphatic index	GRAVY	Localization predicted
CcGASA1	12.25	9.48	A(11.5%), C(10.6%), L(8.8%)	33.50	64.87	-0.165	chlo, extr, vacu
CcGASA2	12.44	8.75	L(12.1%), C(11.2%), G(11.2%)	28.19	84.74	0.011	extr, vacu, ER
CcGASA3	11.42	9.28	C(11.7%), K(8.7%), P(7.8%)	42.68	57.77	-0.095	extr, chlo, nucl, mito
CcGASA4	11.76	9.02	C(12.1%) , R (10.3%), L (8.4%),T(8.4%)	26.38	71.03	-0.079	extr, vacu
CcGASA5	12.32	9.01	C(11.5%), R(9.7%); L(8.0%); T(8.0%)	26.47	69.03	-0.123	extr, vacu, chlo
CcGASA6	9.68	8.62	C(13.6%), K(11.4%), S(9.1%)	28.36	63.07	-0.086	extr, vacu
CcGASA7	15.86	9.59	C(9.1%), A(8.4%), S(8.4%)	67.18	81.19	-0.122	chlo, extr
CcGASA8	11.02	8.66	A(10.6%), C(11.5%), S(9.6%)	46.75	77.02	0.121	extr
CcGASA9	9.38	8.79	C(13.8%), T(10.3%), G(9.2%)	47.05	38.16	-0.574	chlo, mito, cyto
CcGASA10	10.42	9.27	C(12.6%), S(11.6%), L(10.5%), K(10.5%)	49.34	69.79	-0.093	extr, vacu
CcGASA11	21.78	9.63	P(25.7%), T(12.6%), K(8.7%)	69.01	60.10	-0.398	extr, chlo, vacu
CcGASA12	11.74	9.29	C(11.3%), K(11.3%), G(9.4%)	40.13	51.60	-0.263	extr, vacu
CcGASA13	9.67	8.70	C(14.8%), K(12.5%), G(6.8%), L(6.8%), P(6.8%), S(6.8%)	40.22	59.77	-0.242	extr, golg
CcGASA14	7.78	8.75	C(17.1%), K(15.7%), P(10.0%)	51.43	25.14	-0.863	—
CcGASA15	7.71	8.04	C(17.1%), E(10.0%), K(10.0%)	46.37	25.14	-0.791	—
CcGASA16	10.43	9.20	C(12.5%), P(10.4%), G(9.4%), T(9.4%)	57.25	30.52	-0.750	chlo, nucl, mito, plas
CcGASA17	9.14	9.15	C(14.8%), K(11.1%), G(8.6%), P(8.6%), Y(8.6%)	50.33	34.94	-0.668	chlo, nucl, extr, mito, cyto
CcGASA18	12.86	9.19	C(10.2%), P(8.5%), S(8.5%)	47.72	56.19	-0.307	extr, vacu, golg

*MW* molecular weight (kDa), *pI* isoelectric point, *GRAVY* grand average of hydropathicity. Hydrophilic is represented by negative value, hydrophobic is represented by positive value. *A* Ala, *C* Cys, *E* Glu, *G* Gly, *K* Lys, *L* Leu, *P* Pro, *R* Arg, *S* Ser, *T* Thr, *Tyr* Y, *chlo* chloroplast, *mito* mitochondria, *cyto* cytoplasm, *extr* extracellular, *vacu* vacuoles, *nucl* nucleus, *golg* golgiosome, *plas* plastid, *ER* endoplasmic reticulum

The WOLF PSORT II program predicted that the citrus CcGASA family proteins were mostly extracellular, or vacuole- and chloroplast-localized. A few of them might be located in endoplasmic reticulum, nucleus, mitochondria, cytoplasm, plastid and golgiosome (Table 2). The 3D model prediction, with a confidence of more than 99.9%, showed that all of them were relatively flexible for possessing random coils (Supplementary Fig. S1). As can be seen, a small α helix was located at the end of the N-terminal, which was connected next to a larger α helix, but the β-strand was only present on two of them, CcGASA7 and CcGASA17 (Supplementary Table S3). The transmembrane topology analysis showed that there was at least one transmembrane helix on CcGASA1, 3, 4, 5, 7, 8,10, and 12 (Supplementary Fig. S2).

### Phylogenetic relationship of the GASA proteins

**Fig. 3:**
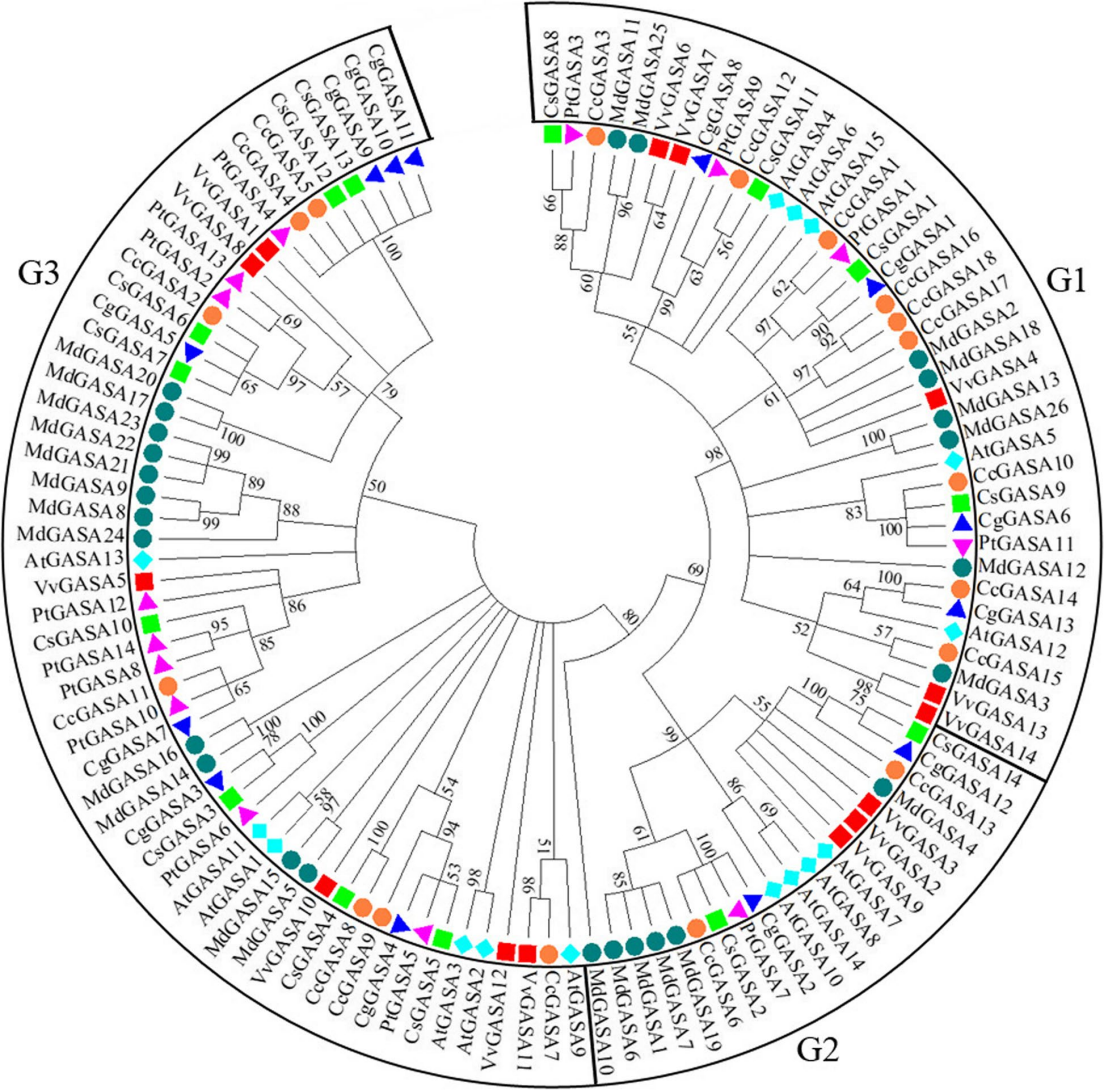
Phylogenetic tree of citrus GASA protein and Arabidopsis AtGASA, apple MdGASA and grape VvGASA. Different groups (G1, G2, and G3) are located in different branches. Protein name: orange-colored dots represent *Citrus clementina* CcGASA proteins, green-colored squares represent *Citrus sinensis* CsGASA proteins, blue-colored squares represent *Citrus maxima* CgGASAs, purple-colored squares represent *Poncirus trifoliata* PtGASAs, blue-colored rhombuses represent *Arabidopsis* AtGASAs, dark green dots represent apple MdGASAs, and red-colored squares represent grape VvGASA proteins. The number near the branch indicates the bootstrap value

An unrooted NJ phylogenetic tree was established by aligning all GASA protein sequences from citrus (56), *Arabidopsis* (15), apple (26) and grape (14) (Fig. 3, Supplementary Table S4). Three branches, G1, G2, G3, were clearly shown on the tree. For citrus CcGASA proteins, CcGASA1, 3, 10, 12, 14, 15, 16, 17 and 18 were clustered with G1, CcGASA2, 4, 5, 7, 8, 9 and 11 were grouped with G3, whereas CcGASA6 and CcGASA13 were classified with G2. Clearly, all the six species analyzed contained homologs of the three branches.

**Fig. 4:**
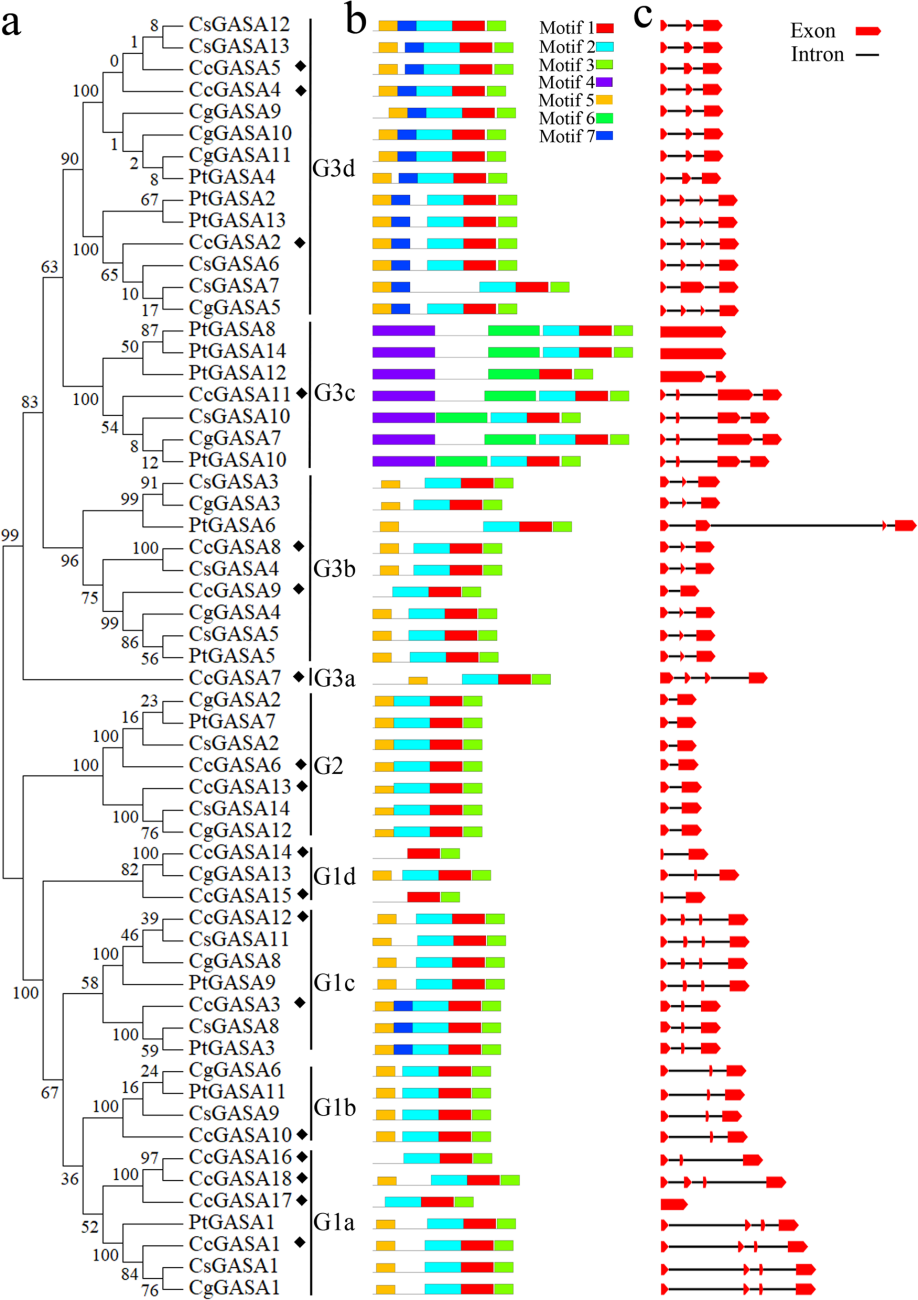
Phylogenetic relationships, exon-intron pattern and group designations in CcGASA proteins from citrus. **a** the neighbor-joining (NJ) tree based on the complete protein sequences of CcGASA. The tree shows the 6 phylogenetic groups (G1a-d, G2, G3a-d). **b** Conserved region analysis of CcGASA proteins. The different colors of boxes denote different motif numbers. The length of box indicates motif length. **c** the gene structure is presented by exon (red boxes) and intron (black line between the red boxes)

The phylogenetic tree for all citrus GASA proteins was shown in Fig. 4a. A total of 7 conserved protein motifs could be identified from the citrus GASA proteins analyzed (Fig. 4b). They were represented by CLRACGTCCARCLCVPPGTYGNKEVC (motif1), SGYTRGLLQSIDCGGLCAARCSLHSRPNP (motif2), CYTBMTTKGGKPKCP (motif3), MAFRAALLLLATLLLVSTSVLSNNEEEYLLEKDTTYPKTPVPAPAPPKAP (motif4), MASRVFLLLSJLLFC (motif5), PTVTPAPPLKPPTTYPPPVKPPTTTPPPVTPPKTAPAPQVP (motif6) and IAVIENQDTQRGZEV (motif7), respectively. It was found that the three motifs, motif1, motif2 and motif3, were universally presented on every member analyzed. Motif5 was missing only in G3c and while motif4 and 6 were only found in the same G3c. Motif7 was appeared only on G3d and on three members of G1. Apparently, the branching of the phylogenetic tree was related to the differences in the arrangement of these motifs. For example, the G2 group members contained 4 closely spaced motifs, motif1, motif2, motif3 and motif5. Similarly, most of the G1 members also contained the same 4 motifs but unlike G2, their motif5 and motif2 was interrupted by insertion of motif7 or a non-motif spacer. Clearly, the citrus G3 members were relatively more diverse than those of G1 and G2 as shown by their motif compositions and motif arrangements, and thus could be further classified into 4 subgroups G3a-d (Fig. 4a). The G1group could also be sub-classified into four sub-groups, G1a-d, Some citrus CcGASA proteins should be structurally or functionally impaired for missing one or two motifs as compared to their respective group members. For instance, motif2 and motif5 were deleted from CcGASA14 and 15, and motif5 was missing in CcGASA9, 16 and 17. The arrangements of exons and introns were relatively conserved in the same group (Fig. 4c). Only one intron was found in G2 while 2 to 3 introns were presented on most of the G1 and G3 group members. Uniquely, two trifoliate orange genes, *PtGASA8* and *14*, and one citrus gene, *CcGASA17*, did not contain any intron.

### *CcGASA* promoters and their possible activators

**Fig. 5:**
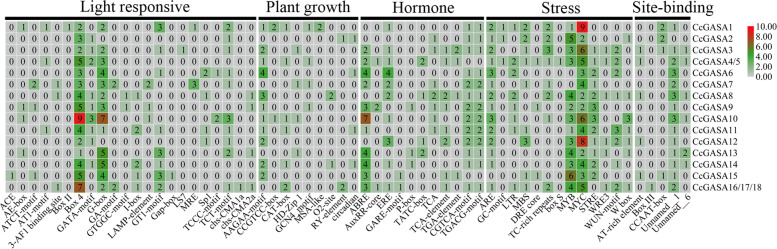
Analysis of cis-elements in *CcGASA* genes. Grey indicates the absence of cis-elements upstream of the *CcGASA* gene. The different colour represents the number of cis-elements upstream of the *CcGASA* gene. The redder of the colour, the greater number of the cis-element

The cis-regulatory elements identified on the promoters of *CcGASA* genes were listed in Fig. 5 and Supplementary Table S5. It was shown that a large number of the elements were stress-related, such as ARE (antioxidant response element), GC-motif (enhancer-like element involved in anoxic specific inducibility), LTR (low-temperature responsiveness), MBS (drought-inducibility), DRE core (cold- and dehydration-responsiveness), TC-rich repeats (defense and stress responsiveness), box S (elicitation, wounding and pathogen responsievness), MYB(abiotic element), MYC(abiotic element), STRE (stress response element), WRE3 (wound-response element 3), WUN-motif (wound responsiveness) and W box (wounding and pathogen responsive). Notably, 2 to 9 MYCs were present on all *CcGASA* promoters. The ARE, essential for the anaerobic induction, was present on 17 *CcGASA* promoters but not on the *CcGASA15* promoter. Hormone responsive elements were also abundant, including ABRE that is responsive to ABA, P-box, GARE-motif and TATC-box that are responsive to GA, AuxRR-core and TGA-element that are responsive to AUX, ERE that is responsive to ET, TCA and TCA that are responsive to SA, and TGACG-motif and CGTCA-motif that are responsive to MeJA. Light response cis-elements, including 3-AF1 binding site, ACE, AE-box, ATCT-motif, AT1-motif, Box II, Box 4, GATA-motif, G-box, GA-motif, GTGGC-motif, I-box, LAMP-element, GT1-motif, Gap-box, LS7, MRE, Sp1, TCCC-motif, TCT-motif, chs-CMA1a and chs-CMA2a, were also frequently found on *CcGASA* promoters. In addition, plant growth and development associated cis-elements, including meristem-specific expression elements, CCGTCC-box and CAT-box, plant seed and shoot development-related RY-element, circadian control element, MSA-like cell cycle control element, and the palisade mesophyll cells differentiation element HD-Zip 1, were also identified on *CcGASA* promoters.

**Fig. 6:**
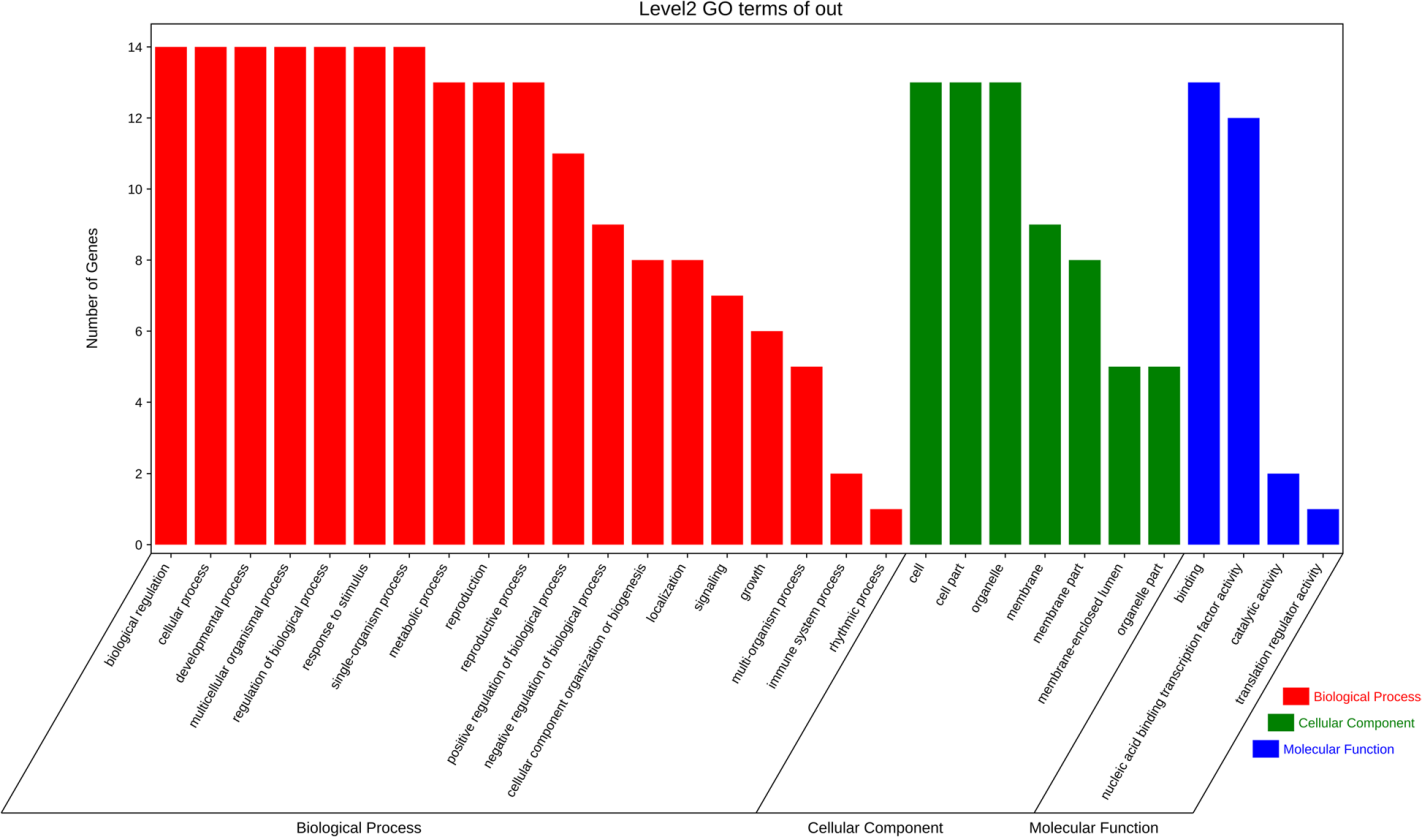
GO enrichment analysis results of predicted TFs. Red bar indicates GO term related to biological process; green bar indicate GO term related to cellular component; Blue bar indicate GO term related to molecular function

The potential transcriptional regulatory network of the *CcGASA* gene family was analyzed. It was shown that the TFs that might bind to the above-mentioned cis-elements were numerous (Supplementary Fig. S3 and Table S6). Most of the TFs were ERF, MYB and MIKC_MADS, for 23, 12 and 9 of them were respectively identified in the study. In addition, 5 Dofs, 4 ARFs, 4 C_2_H_2_s, and 4 HSF were also predicted to be the activators of the *CcGASA* genes. Functionally, these TFs were mostly associated with abiotic and biotic stresses such as pathogen attacks, heat shock and drought stresses. In addition, some TFs including ERF (Ciclev10009361m.g), ERF (Ciclev10025816m.g), ARF (Ciclev10000183m.g, Ciclev10011065m.g, Ciclev10014391m.g, Ciclev10030860m.g), GRAS (Ciclev10017466m.g), and C2H2 (Ciclev10002297m.g) that are responsive to plant hormones are known to be involved in plant growth and development regulations. Tissue specific TFs were also identified, including the lateral organ boundaries transcription factor LBD (Ciclev10024416m.g) and the root specific transcription factor NAC (Ciclev10010579m.g). Detailed GO function enrichment analysis showed that these putative transcription factors were mainly enriched in the cellular process (GO:0009987), multicellular organismal process (GO:0032501), developmental process (GO:0032502), single-organism process (GO:0044699), regulation of biological process (GO:0050789), biological regulation (GO:0065007), metabolic process (GO:0008152) and response to stimulus (GO:0050896) (Fig. 6).

### Expression of *CcGASAs* under treatments of Xcc and plant hormones

**Fig. 7:**
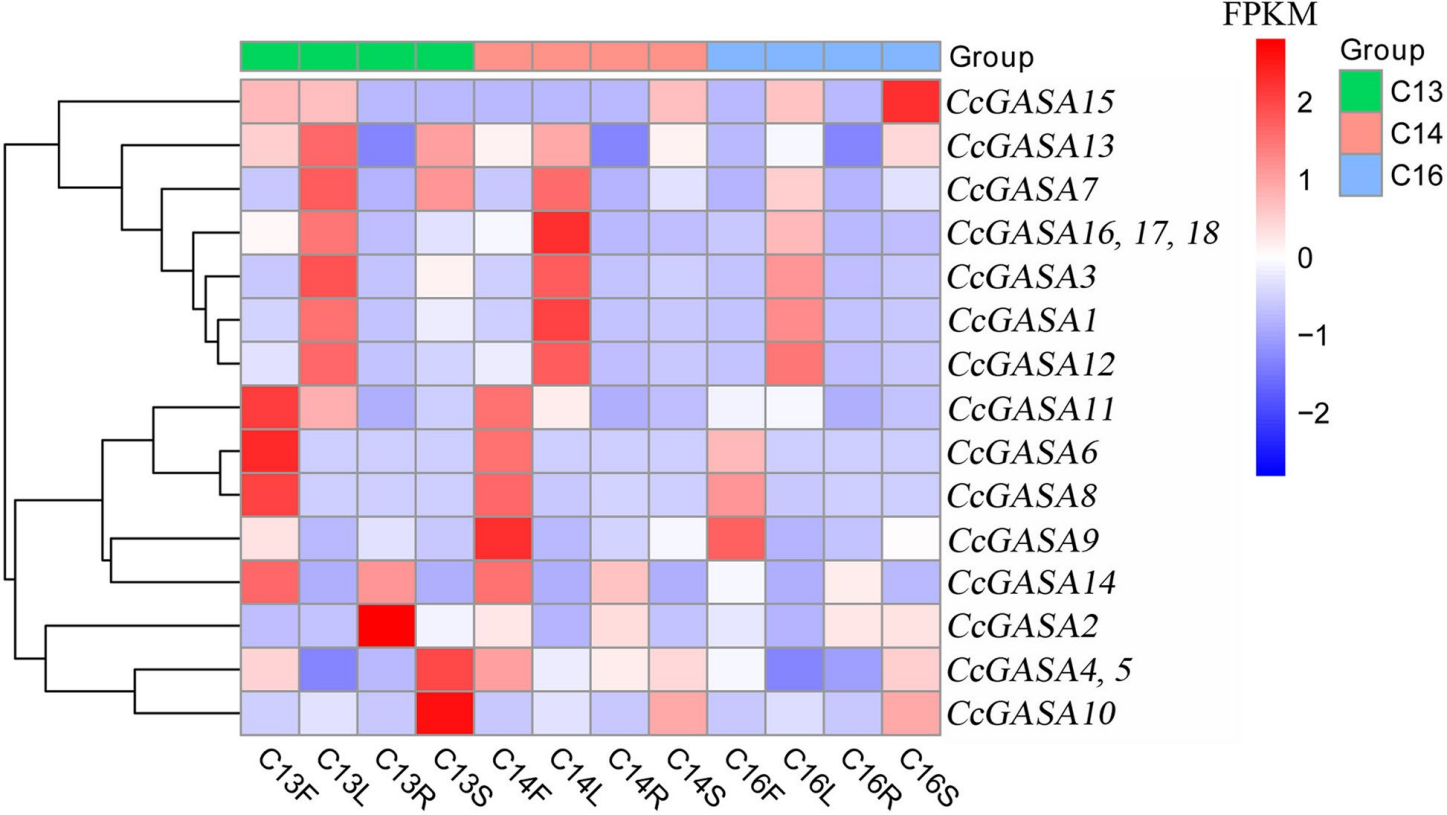
Transcript analysis of *CcGASAs* in fruits (F), leaves (L), roots(R) and stems (S) of citrus. The C13, C14 and C15 represent three independent *C. clementina* plants. The expression abundance of genes is showed by the FPKM value, and from blue to red, the higher the expression level of genes

The basal expression of all *CcGASA* genes was analyzed in different organs of *C. clementina* (Fig. 7). The results showed that *CcGASA1*, *CcGASA3*, *CcGASA7*, *CcGASA12*, *CcGASA16*, *CcGASA17* and *CcGASA18* were preferably expressed in leaves. *CcGASA6*, *CcGASA8*, *CcGASA9* and *CcGASA11* were highly expressed in fruits. *CcGASA2* was mainly expressed in roots. *CcGASA4*, *CcGASA5* and *CcGASA10* were most abundantly expressed in stems. *CcGASA14* was predominantly expressed in fruits and, to a lesser extent, in roots. Similarly, *CcGASA13* was most highly expressed in leaves and next highly expressed in stems.

**Fig. 8:**
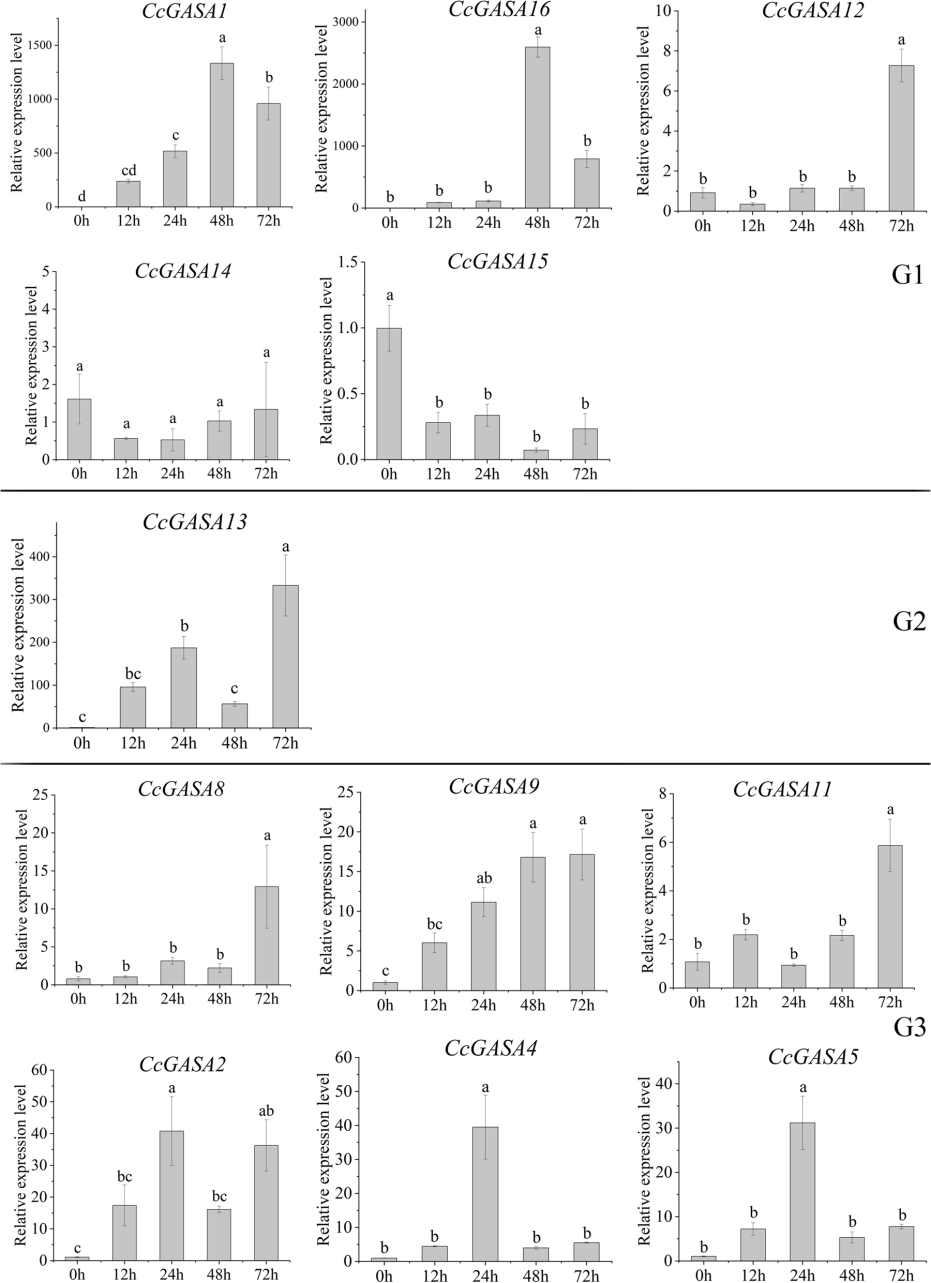
Relative expression level of *CcGASAs* in citrus leaves inoculation with Xcc. Mean±standard error of three replicates is shown. Different lowercases letters (**a-e**) on the bars indicate statistically significant differences (*P*<0.05) based on Duncan’s LSD multiple range test

The inducibility of the *CcGASA* genes by Xcc was investigated. As shown in Supplementary Fig. S4, the detached *C. clementina* leaf explants developed water-soaked symptoms and white spots around the pinholes within 7d following inoculation with Xcc, the citrus canker pathogen. As expected, no such symptom was observed on control leaves. The qRT-PCR analysis showed that the *CcGASA* genes were mostly induced by Xcc (Fig. 8). Ten of the genes, *CcGASA1*, *2*, *4*, *5*, *8*, *9*, *11*, *12*, *13*, *16* were highly induced by Xcc, as exemplified by peak induction of more than 1000-fold for *CcGASA1* and *CcGASA16*, and 300-fold for *CcGASA13*. Three clear patterns, gradual induction, later-stage induction and middle-stage induction, were observed. Gradual induction occurred to *CcGASA1*, *2*, *9*, *13*. Later-stage induction occurred to 4 genes, *CcGASA8*, *11*, *12*, *16*, for their expression was mostly peaked at 72 h. The expression of two genes, *CcGASA4* and *CcGASA5*, was peaked at 24h, i.e., in the middle of the treatment. Two genes, *CcGASA14 and CcGASA15*, was shown to be moderately repressed or not induced by Xcc infection (Fig. 8).

**Fig. 9:**
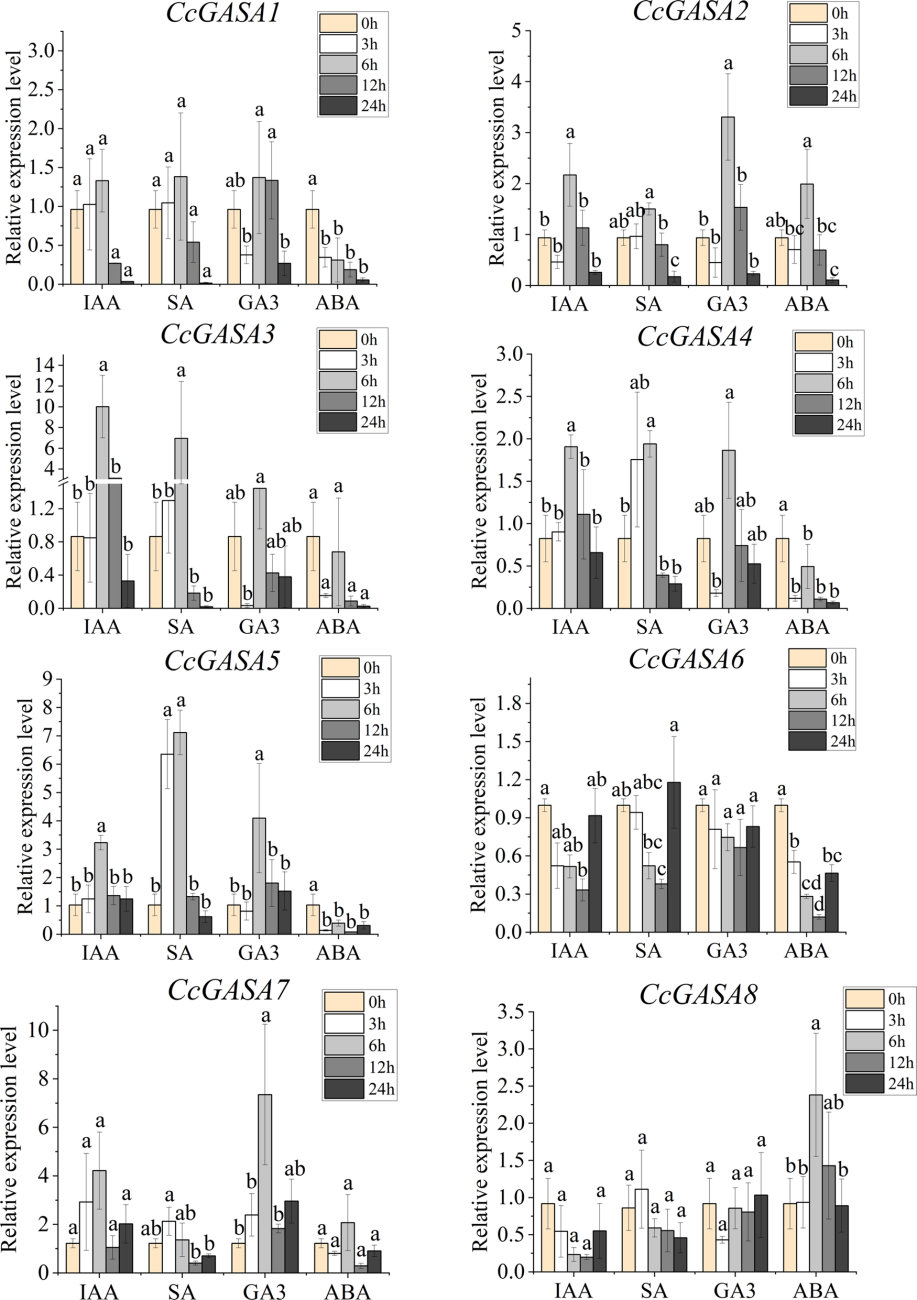
The expression abundance of *CcGASA1-8* during IAA, SA, GA_3_ and ABA treatment. Data are mean ± SE of 3 qRT-PCR experiments and 3 biological replicates. Different lowercases letters (**a-c**) on the bars indicate statistically significant differences (*P*<0.05) based on Duncan’s LSD multiple range test

The expression patterns of *CcGASA* genes were found to be modulated by hormone treatments (Fig. 9, Supplementary Figs. S5 and S6). It was shown that *CcGASA3*, *4*, *5*, *10*, *11*, *12* and *CcGASA14* were significantly up-regulated by IAA treatment. The highest up-regulation, about 12-fold, was shown by *CcGASA3*. The second highest induction, about 10-folds, was shown by *CcGASA12. CcGASA11* was up-regulated by about 9-folds. In contrast, *CcGASA13*, *15*, *16* and *CcGASA17* were significantly down-regulated by IAA. Under SA treatment, the expression of *CcGASA2*, *3*, *4*, *5*, *10*, *11*, *12 and CcGASA13* followed an inverted V-shape pattern whereas *CcGASA16* and *CcGASA17* were significantly down-regulated. Under GA_3_ treatment, the expression of *CcGASA1*, *2*, *11* and *CcGASA12* was increased at the beginning, peaked in the middle and subdued at the end of the treatment while the expression of *CcGASA3*, *4* and *CcGASA5* were decreased at 3h, but reversed to increase at 6h and 12h, and then decreased again at the end of the treatment. Notably, 3 genes, *CcGASA7*, *10* and *CcGASA15* were always up-regulated whereas 3 other genes, *CcGAS13*, *16* and *CcGASA17* were always down-regulated under GA_3_ treatment. Under ABA treatment, *CcGASA8*, *10* and *14* were up-regulated, *CcGASA2*, *7* and *9* were initially increased and then decreased, whereas the other remaining genes were always down-regulated.

### Evolution of citrus *CcGASA* genes

The possible tandem duplication events were inferred from analyzing the sequences of all *CcGASA* genes according to Holub et al. [21]. It was shown that a recent whole gene duplication event might be responsible for the cluster of two genes, *CcGASA8* and *CcGASA9,* on scaffold 5. A more ancient whole gene duplication event should have generated another cluster on scaffold 9, *CcGASA14* and *CcGASA15*. Interspersed segmental duplications were also detected across different scaffolds as shown in Fig. S7. They should be responsible for the generation of 4 pairs, *CcGASA2*-*CcGASA5*, *CcGASA3*-*CcGASA12*, *CcGASA5*-*CcGASA11* and *CcGASA6*-*CcGASA13* (Table 3). Ka/Ks ratios were calculated to measure the selection pressures between each of the 6 gene pairs (Table 3). The results showed that the Ka/Ks ratios were all less than 1, suggesting that the duplicates had experienced purifying selection.

**Table 3:** Duplicate information in the *Citrus Clementina* GASA family

Seq 1	Seq 2	Ka	Ks	Ka/Ks	Selection pressure	Gene duplications
Ciclev10002927m.g-CcGASA8	Ciclev10002984m.g-CcGASA9	0.1947	0.4914	0.3962	purifying	tandem
Ciclev10006931m.g-CcGASA14	Ciclev10006668m.g-CcGASA15	0.0910	1.2137	0.0750	purifying	tandem
Ciclev10033115m.g-CcGASA5	Ciclev10022925m.g-CcGASA2	0.2781	1.7066	0.1630	purifying	interspersed
Ciclev10013454m.g-CcGASA12	Ciclev10023012m.g-CcGASA3	0.2924	2.7325	0.1070	purifying	interspersed
Ciclev10012786m.g-CcGASA11	Ciclev10033115m.g-CcGASA5	0.4009	2.5934	0.1546	purifying	interspersed
Ciclev10029695m.g-CcGASA13	Ciclev10002979m.g-CcGASA6	0.2638	1.1030	0.2392	purifying	interspersed

**Fig. 10:**
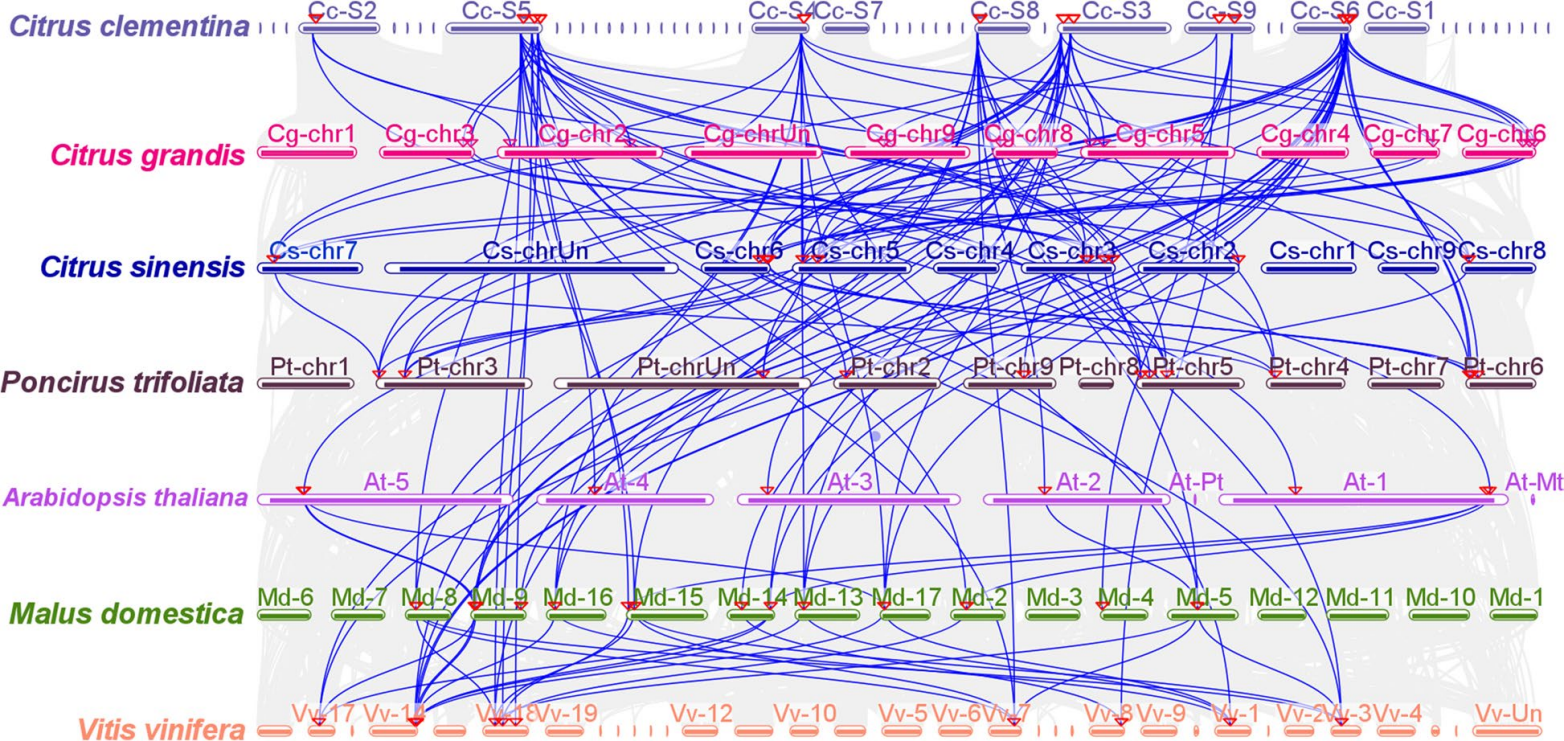
The synteny analysis of *GASA* genes among citrus, *Arabidopsis*, apple and grape. According to the chromosomes of citrus, *Arabidopsis*, apple and grape, the relative positive positions are depicted. Gray lines in the background indicate the collinear blocks within citrus, *Arabidopsis*, apple and grape, while the blue lines highlight the syntenic *GASA* gene pairs

A synergy analysis of the orthologous *GASA* genes from *C. clementina*, *C. sinensis*, *C. maxima*, *P. trifoliata*, *A. thaliana*, *M. domestica* and *V. vinifera* genomes identified 96 collinearity events between *C. clementina* and the other six species. It was found that 6, 12, 11, 11, 11 and 10 *CcGASA* genes had synonyms in *A. thaliana*, *C. maxima*, *C. sinensis*, *P. trifoliata*, apple, and grape, respectively (Fig. 10 and Supplementary Table S7). In addition, we found that *CcGASA8*, *13*, *12* were collinearity with the *GASA* genes of the other 6 species, indicating that these 3 genes play a very important role in the expansion of the GASA family.

### Protein interaction network of CcGASA proteins

The possible interactions between the 18 CcGASAs and other proteins were analyzed. As shown in Supplementary Fig. S8, only 8 CcGASAs were predicted to have 10 functional partners which were Cellulose synthase (XP_006439113.1), ATPase ASNA1 homolog (XP_006445186.1) and uncharacterized proteins, XP_006440347.1, XP_006440529.1, XP_006427852.1, XP_006443889.1, XP_006437427.1, XP_006437429.1, XP_006430408.1 and XP_006433793.1. The remaining 10 CcGASA proteins (CcGASA1, 2, 4, 5, 9, 14, 15, 16, 17, 18) were not successfully predicted to have a functional partner. CcGASA11 might have a potential relationship with CcGASA3 and CcGASA12 respectively. Both CcGASA12 and CcGASA3 were predicted to interact with XP_006445186.1 and XP_006445186.1 that participate in the peroxisome pathway and the proteasome pathway. The CcGASA6, CcGASA8 and CcGASA13 could respectively interact with XP_006443889.1. CcGASA10 was predicted to interact with 5 proteins, XP_006430408.1, XP_006440529.1, XP_006437427.1, XP_006437429.1 and XP_006439113.1. CcGASA7 was predicted to interact with XP_006427852.1.

## Discussion

The low molecular-weight GASA proteins have been identified in different plants, and a large number of functional studies have shown their crucial roles in regulating plant growth, development and defense against pathogens [46]. However, the detailed regulation mechanisms through which GASAs operate have not yet been established. We previously found that a *Citrus GASA* gene was induced by *Citrus tristiza virus* infection [54]. To go a step deeper in understanding the role of the gene and, in a broad sense, other *GASA* genes in citrus, we set out to conduct a genome-wide bioinformatics study on the *C. clementina GASA* gene family.

In this study, 15 *CcGASA* genes were identified from the whole-genome sequence of *C. clementina*, including the gene previous reported by us [54]. Their genomic DNA and deduced protein sequences were compared with each other and with their homologous genes from other plant species, allowing the establishment of the inter-genomic and the intragenic phylogenetic trees. Detailed analysis on gene structures, cis-elements, chromosomal locations was performed. Primary sequences, physiochemical properties, subcellular localizations, 3D structures, transmembrane domains, motifs of the proteins were also analyzed. In addition, evolution of the *CcGASA* genes was investigated and possible duplication events were thus identified. Collinear relationships were revealed between the *CcGASA* genes and those from other citrus species, arabidopsis, apple and grape. The transcription factors possibly binding to *CcGASA* promoters, and the proteins possibly interacting with CcGASAs were also predicted and their associated functions were analyzed.

Our results indicated that CcGASA might be involved in plant defense processes. Firstly, these small hydrophilic and unstable proteins were mostly predicted to be in the extracellular space (Table 2), strongly indicating their association with plant cell walls that constitute the first line of defense in plants [44]. Secondly, their involvement in stress regulation was also suggested by the presence of many stress responsive *cis*-acting elements on their promoters (Fig. 5), such as ABRE involved in ABA-regulated osmotic stress [25], CGTCA-motif and TGACG-motif involved in the regulation in seed germination, senescence, and stress responses [10], ERE required for the expression of most ethylene-induced genes [23]. In addition, anaerobic (ARE and/or GC-motif), abiotic (MYB, MYC and/or STRE), low temperature (LTR), drought (MBS/DRE core), pathogens (TC-rich repeats/box S/W box) and wounding (WUN-motif/W box) induced/responsive elements were also very abundant. Furthermore, analysis for TFs interacting with these elements also indicated that many of the TFs were associated with stress responses (Supplementary Fig. S3). Moreover, the possible involvement of some CcGASA proteins in defense was also shown by their predicted interactions with defense-related proteins (Supplementary Fig. S8). Thirdly, the expression of the *CcGASAs* was induced by treatments of stress-related hormones and citrus canker bacterium Xcc (Figs. 8 and 9 and Supplementary Figs. S5 and S6).

The *GASA* genes have also been known to play important roles in plant growth and development [34]. In this regard, the nature of some cis-elements found on their promoters (Supplementary Table S5), such as tissue specific expression elements, AAGAA-motif (driving endosperm-specific negative expression), CAT-box (specifying meristem expression) and many light responsive elements, among others, implied that the *CcGASA* genes should indeed play important roles in the growth and development of citrus. Moreover, GA (GARE-motif/P-box/TATC-box), SA (TCA/TCA-element) and auxin (AuxRR-core/TGA-element) responsive elements were found in the promoter regions of most *GASA* genes. GO analysis also showed that some TFs identified by bioinformatics method in the study are associated with plant development (GO: 0032502), biological regulation (GO: 0050789 and GO: 0065007), responses to stimuli (GO: 0050896) (Fig. 6). That several flowering-related TFs were identified indicated that the citrus *CcGASA* genes or at least some of them should play a role in reproduction processes in *Citrus,* which is similar to *Arabidopsis AtGASA4*, a flowering promotion gene [38], and *AtGASA5*, a flowering delaying gene [58]. It should be noted that both *Arabidopsis* genes are actually involved in GA-mediated flowering.

The above notions that CcGASA proteins should play multiple roles in citrus were further supported by phylogenetic classification results. As shown in Fig. 4, the proteins were classified into 3 large groups and the classification was apparently related to their motif compositions (Fig. 4b) and primary sequences (Fig 2). It has long been known that structure and function of a protein is interrelated [20]. Thus, variations occurred to GASA protein structures should have allowed them to evolve different functions although they still share the highly conserved GASA domain. This could be exemplified by two G1d genes, *CcGASA14* and *15* that have lost two motifs in their primary sequences (Fig. 4b). The possible consequence of such a large structural variation might be far-reaching, i.e., the pathogen inducibility they shared with other paralogs in the same G1 group was lost forever (Fig. 8). Structural differentiations among GASA proteins should also have resulted in functional differentiations. In this respect, the Xcc induction of G1a members, represented by *CcGASA1* and *CcGASA16*, was 2 and 3 orders of magnitude higher than that of G2 and G3 members (Fig. 8), respectively, which strongly suggested that the G1a members have been specialized to cope with biotic stresses. However, we need to do more investigations to demonstrate this speculation.

The expansion of the *GASA* gene family was shown to be mainly through DNA duplication, either interspersed segmental duplication or tandem duplication [40]. In this study a total of 15 *CcGASA* genes were identified in *C. clementina*. Comparatively, there are about 15, 14, 26, and 37 GASA genes in *Arabidopsis*, grape, apple and soybean, respectively [1, 2, 14]. As can be seen, there are approximately two times more *GASA* genes in apple and soybean than in citrus, grape and *Arabidopsis*. Such a large discrepancy in *GASA* gene numbers between different species could be better explained by that only apple and soybean respectively experienced a recent whole genome duplication (WGD) while the others did not [41, 50]. Similarly, it was found that the *EIN3/EIL* genes were doubled from 4~5 in *Arabidopsis*, tobacco, tomato, rice, peach, mei and strawberry that did not have a recent WGD, to 10 in pear that shared a recent WGD with apple [11, 53, 55]. The Ka/Ks ratios between the 6 paralogous GASA gene pairs were all less than 1 (Table 3), indicating that they have been undergoing a purifying selection rather than a positive or neutral selection. Comparison of the two tandem duplication originated pairs, *CcGASA14 / 15* and *CcGASA8 / 9*, with other *CcGASA* genes revealed that both CcGASA14 and 15 lost two motifs while CcGASA9 lost one motif (Fig. 4b), indicating that the three genes might either undergo degeneration or evolve new functions.

## Conclusions

Eighteen *CcGASA* proteins from the *C. clementina* genome were identified and analyzed in this study, with emphasis on their possible roles in defense in citrus. Results from bioinformatics analysis showed that the members of the gene family have structurally and functionally diverged to different degrees and thus may play different roles in the growth and development of *Citrus*. Experimental evidence showed that the expression of the G1a subgroup members was highly sensitive to bacterial infection, strongly suggesting that they may play an important role in the responses of citrus to biotic stresses.

## Supplementary Information

**Additional file 1: Table S1.** List of CcGASA proteins along-with their protein, CDS and genomic sequence. **Table S2.** Primers used for qRT-PCR analysis. **Table S3.** Secondary structures of CcGASA protein. **Table S4.** Protein, CDS and genomic sequences of *Citrus sinensis*, *Citrus maxima*, *Poncirus trifoliata*, *Arabidopsis*, apple and grape. **Table S5.** Analysis of cis-elements in the *CcGASA* genes. **Table S6.** Sequence information of predicted transcription factors (TF) that interacts with *CcGASA* genes. **Table S7.** The collinearity genelink of GASA family genes in citrus, *Arabidopsis*, apple and grape genomes.

**Additional file 2: Figure S1.** Predicted three- dimensional (3D) structures of CcGASA proteins. Ribbon representation of the structural model obtained by Phyre^2^, illustrating the mainly helical structure, characteristic of the GASA protein fold.

**Additional file 3: Figure S2.** Transmembrane topology analysis of CcGASA proteins. The Y axis represents probability, and the X axis represents the number of amino acid residues. The red peaks indicate the predicted transmembrane helices.

**Additional file 4: Figure S3.** The putative transcription factor regulatory network of the *CcGASA* genes. The transcriptional regulatory network was constructed with the PTRM tool and Cytoscape 3.8 software. The same color represents transcription factors of the same family. For example, pink represents ERF, green represents MYB, and blue represents MIKC_MADS.

**Additional file 5: Figure S4.** *Citrus clementina* leaves inoculated with Xcc.

**Additional file 6: Figure S5.** The expression abundance of *CcGASA9-16* genes during IAA, SA, GA_3_ and ABA treatment. Data are mean ± SE of 3 qRT-PCR experiments and 3 biological replicates. Different lowercases letters (a-c) on the bars indicate statistically significant differences (*P*<0.05) based on Duncan’s LSD multiple range test.

**Additional file 7: Figure S6.** The expression abundance of *CcGASA17* during IAA, SA, GA_3_ and ABA treatment. Data are mean ± SE of 3 qRT-PCR experiments and 3 biological replicates. Different lowercases letters (a-c) on the bars indicate statistically significant differences (*P*<0.05) based on Duncan’s LSD multiple range test.

**Additional file 8: Figure S7.** Chromosomal distribution and synteny analysis of citrus *CcGASA* gene family members. Syntenic regions and chromosomal regions are depicted in different colors.

**Additional file 9: Figure S8.** Putative protein-protein interaction network of CcGASA proteins in *C.clementina*. Colored nodes: query proteins and first shell of interactors. white nodes: second shell of interactors. Empty nodes: proteins of unknown 3D structure. Filled nodes: some 3D structure is known or predicted.

## Data Availability

All data generated or analyzed during this study are included in this published article and its supplementary information files.

## Abbreviations

GASA: Gibberellic Acid Stimulated in Arabidopsis
MCScanX: Multiple Collinearity Scan toolkit
SDs: Segmental duplications
Ka: Non-synonymous
Ks: Synonymous
WGD: Whole genome duplication
