# Supplementary figures and images for: Analysis of CcGASA family members in Citrus clementina (Hort. ex Tan.) by a genome-wide approach
Source: BMC Plant Biol. 2021 Dec 1;21:565. doi: 10.1186/s12870-021-03326-6 (PMC8638133; doi:10.1186/s12870-021-03326-6)

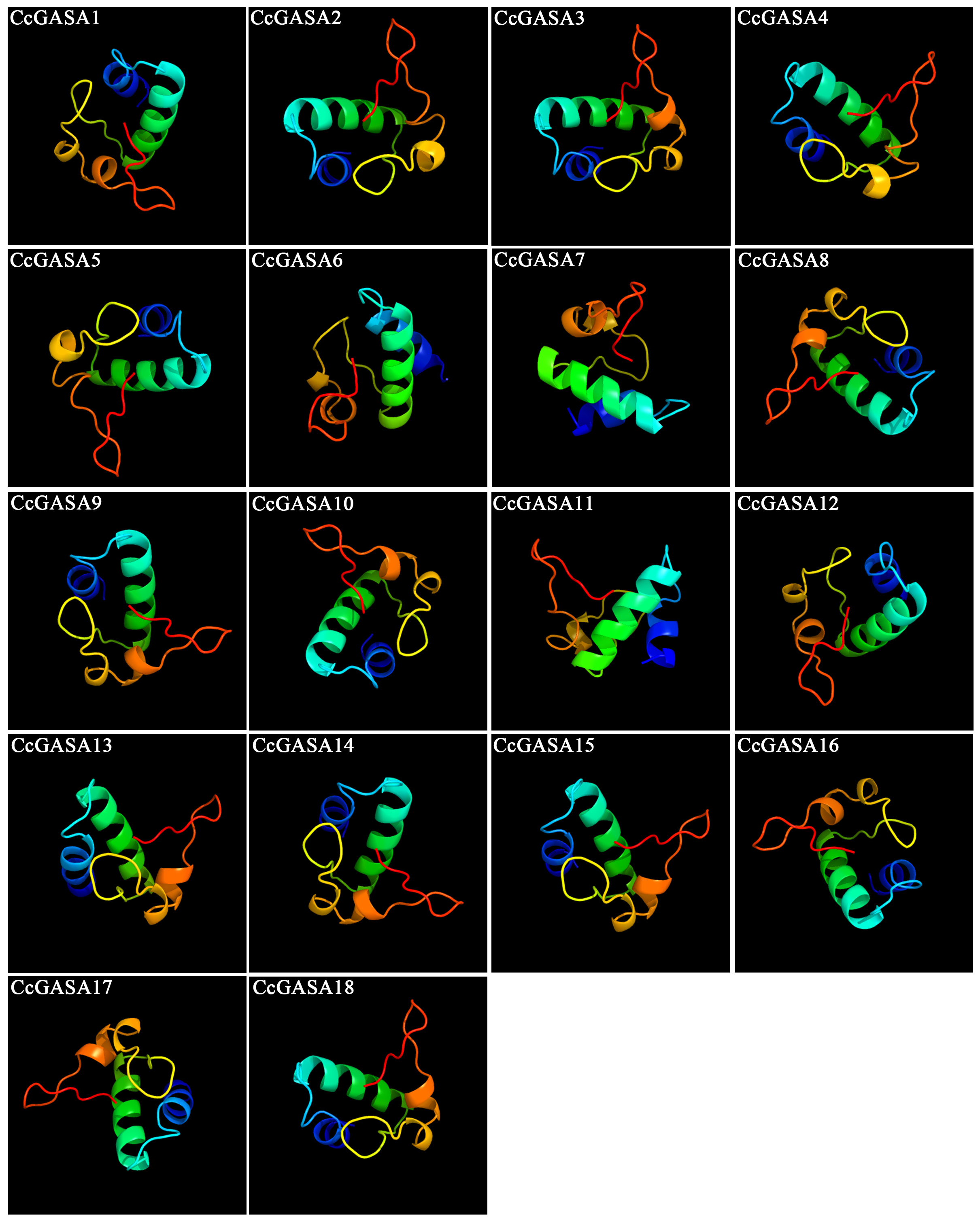

Supplement: Supplementary file 2 — Additional file 2: Figure S1. Predicted three- dimensional (3D) structures of CcGASA proteins. Ribbon representation of the structural model obtained by Phyre2, illustrating the mainly helical structure, characteristic of the GASA protein fold. [file 12870_2021_3326_MOESM2_ESM.jpg]

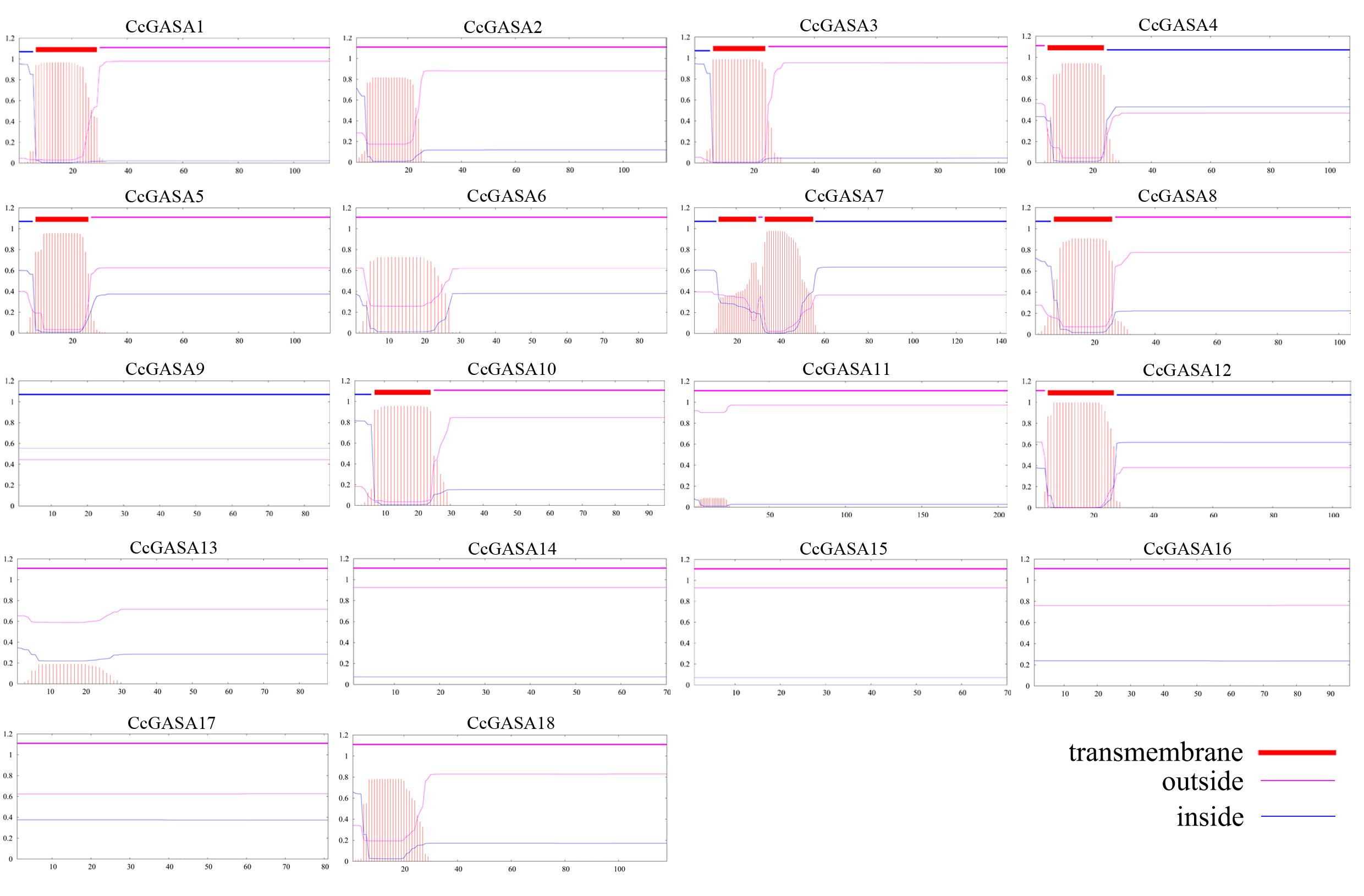

Supplement: Supplementary file 3 — Additional file 3: Figure S2. Transmembrane topology analysis of CcGASA proteins. The Y axis represents probability, and the X axis represents the number of amino acid residues. The red peaks indicate the predicted transmembrane helices. [file 12870_2021_3326_MOESM3_ESM.jpg]

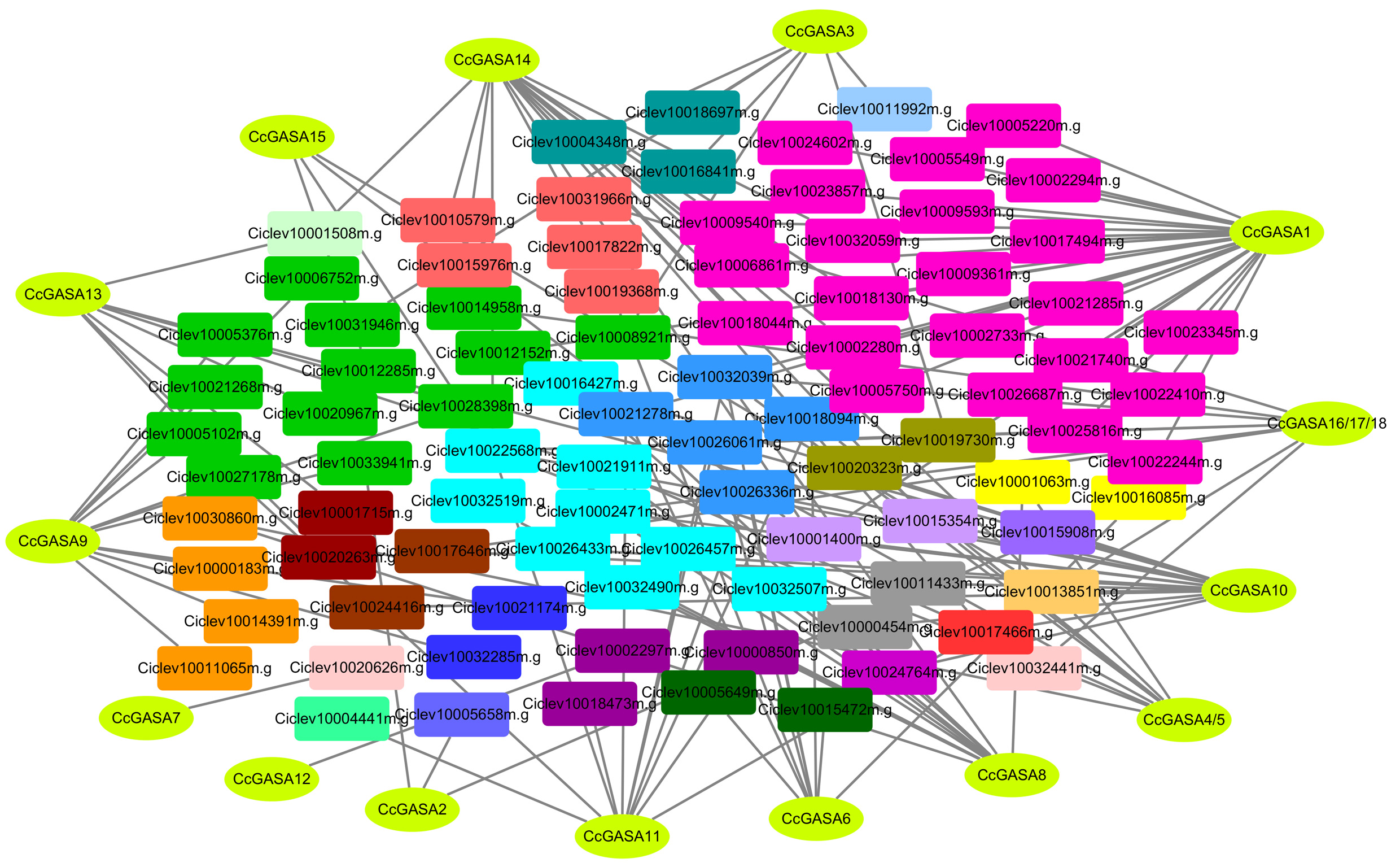

Supplement: Supplementary file 4 — Additional file 4: Figure S3. The putative transcription factor regulatory network of the CcGASA genes. The transcriptional regulatory network was constructed with the PTRM tool and Cytoscape 3.8 software. The same color represents transcription factors of the same family. For example, pink represents ERF, green represents MYB, and blue represents MIKC_MADS. [file 12870_2021_3326_MOESM4_ESM.jpg]

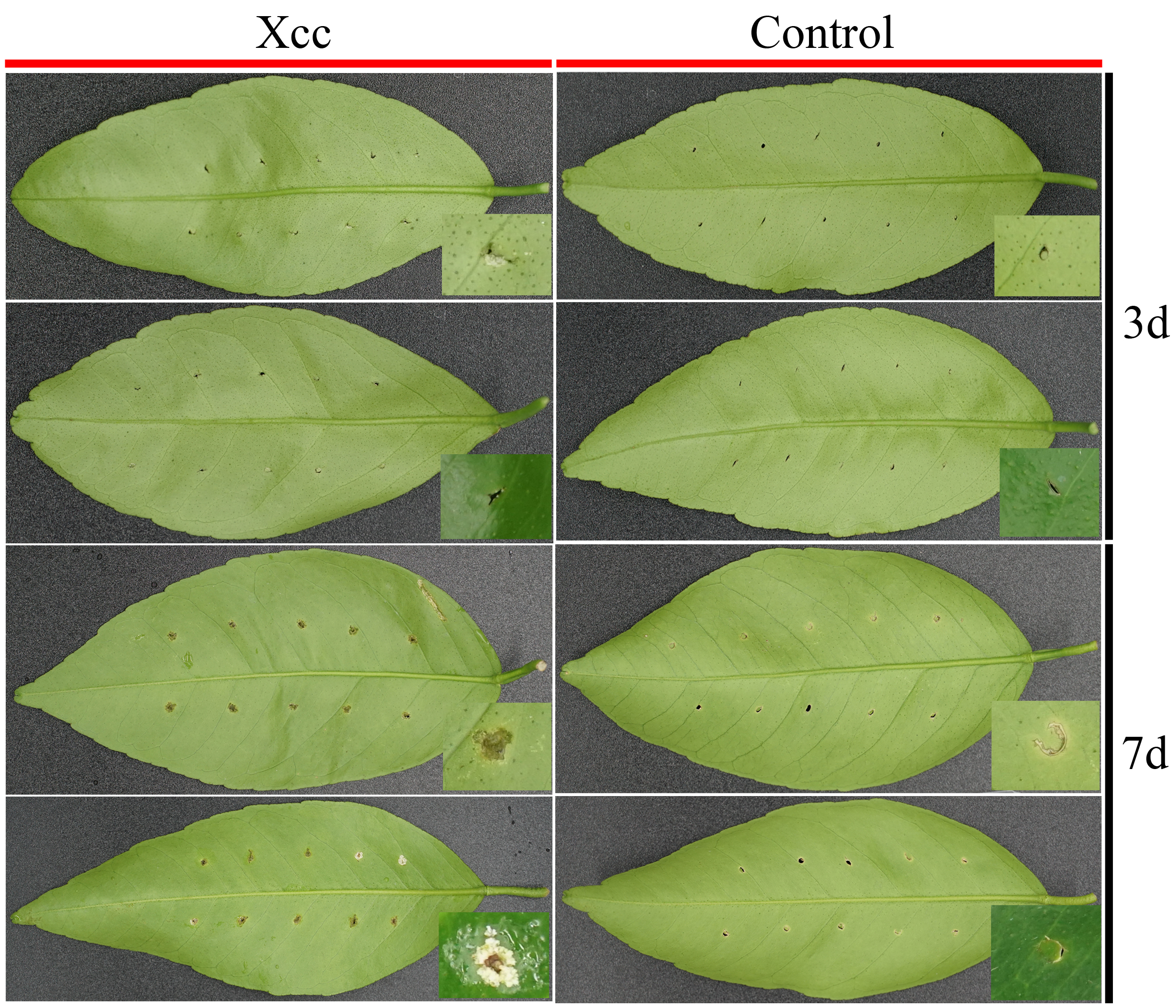

Supplement: Supplementary file 5 — Additional file 5: Figure S4. Citrus clementina leaves inoculated with Xcc. [file 12870_2021_3326_MOESM5_ESM.jpg]

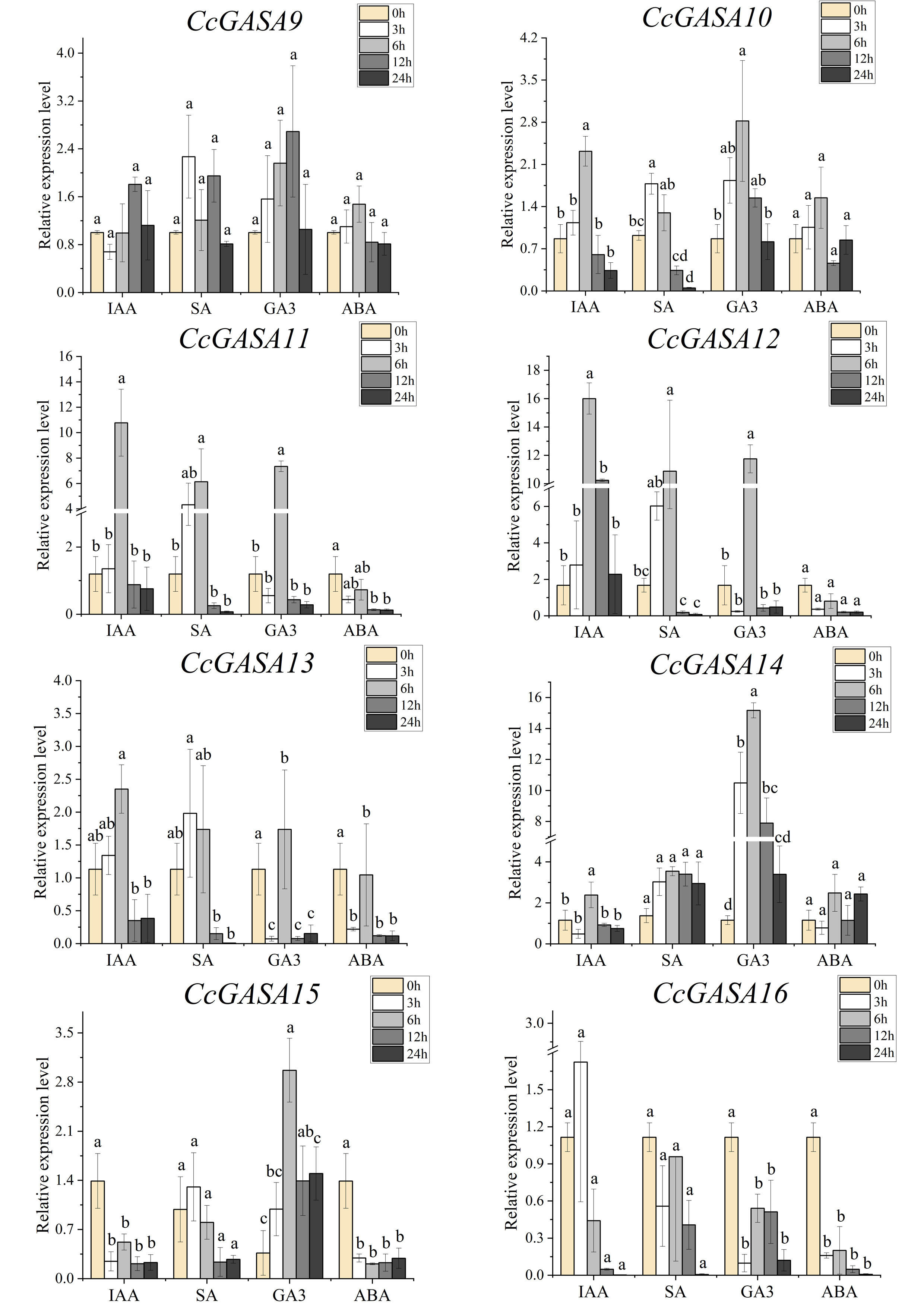

Supplement: Supplementary file 6 — Additional file 6: Figure S5. The expression abundance of CcGASA9-16 genes during IAA, SA, GA3 and ABA treatment. Data are mean ± SE of 3 qRT-PCR experiments and 3 biological replicates. Different lowercases letters (a-c) on the bars indicate statistically significant differences (P<0.05) based on Duncan’s LSD multiple range test. [file 12870_2021_3326_MOESM6_ESM.jpg]

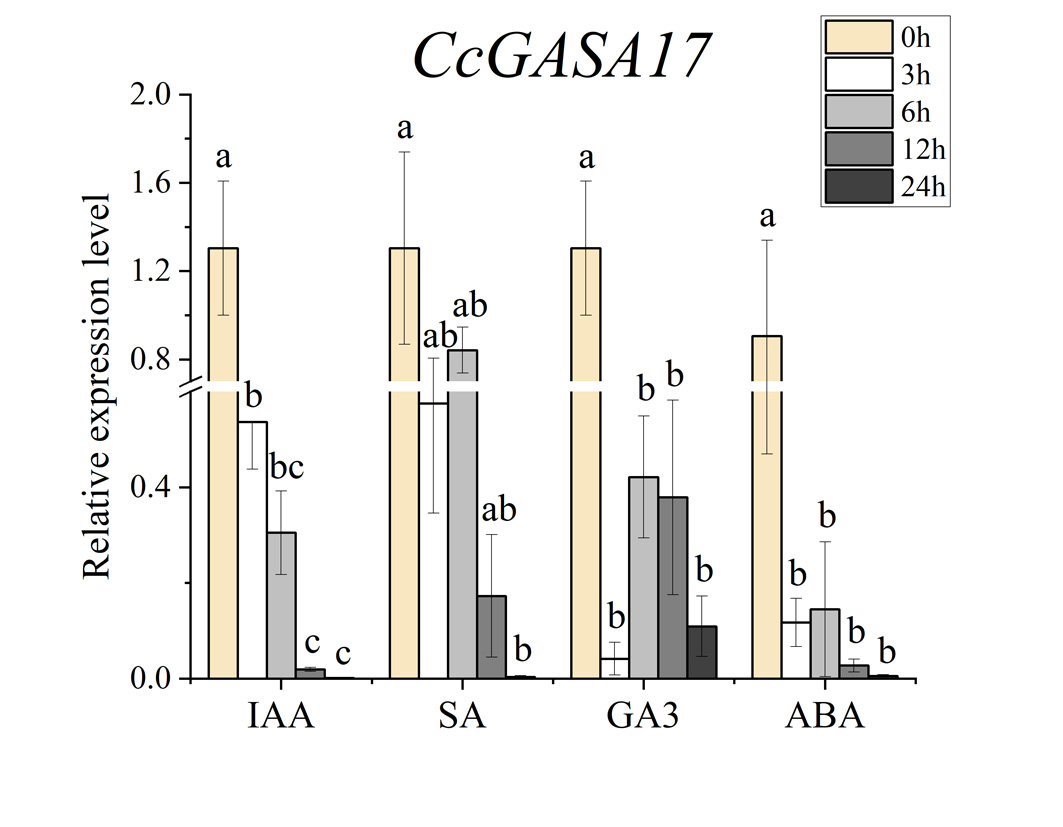

Supplement: Supplementary file 7 — Additional file 7: Figure S6. The expression abundance of CcGASA17 during IAA, SA, GA3 and ABA treatment. Data are mean ± SE of 3 qRT-PCR experiments and 3 biological replicates. Different lowercases letters (a-c) on the bars indicate statistically significant differences (P<0.05) based on Duncan’s LSD multiple range test. [file 12870_2021_3326_MOESM7_ESM.jpg]

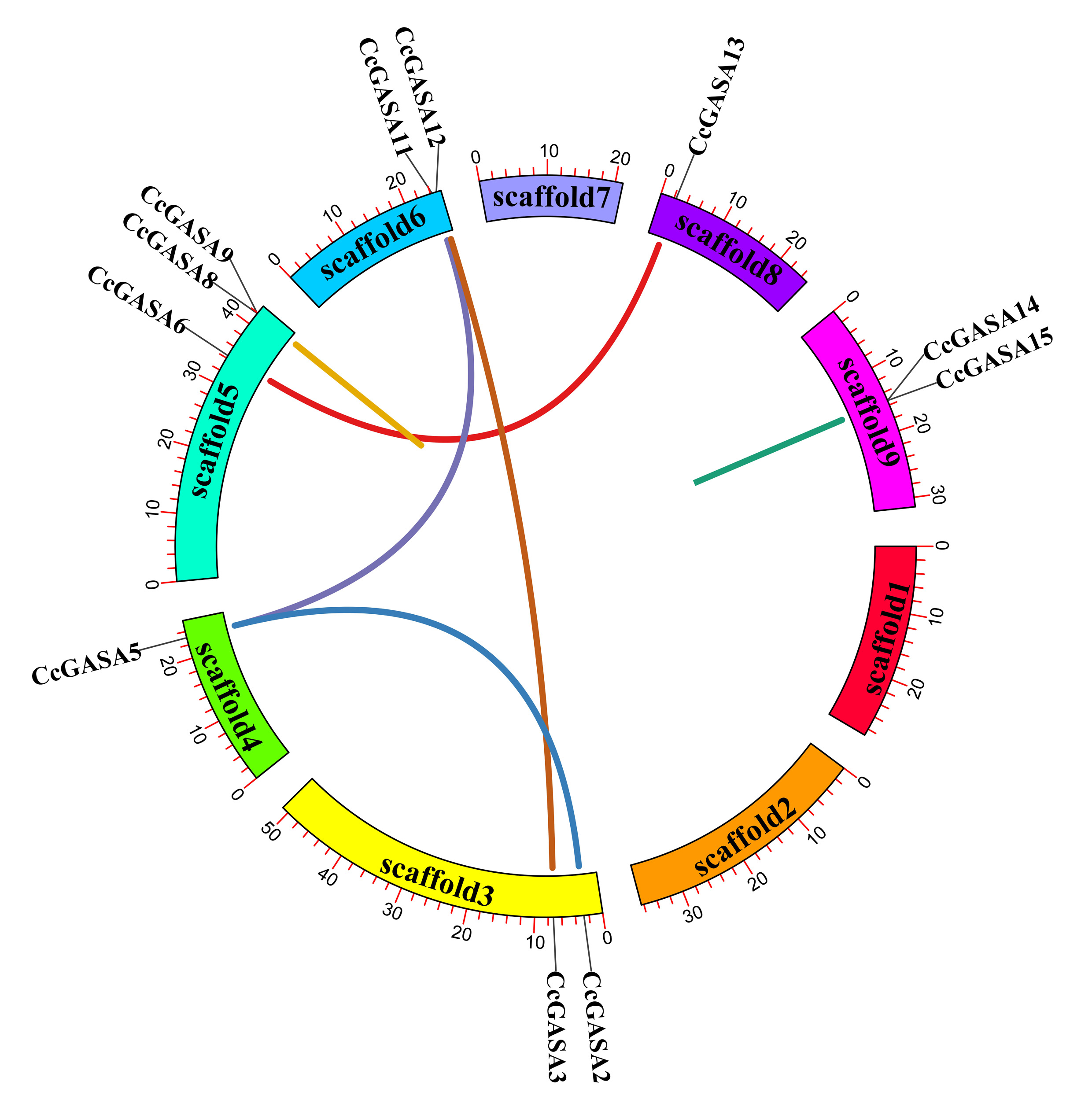

Supplement: Supplementary file 8 — Additional file 8: Figure S7. Chromosomal distribution and synteny analysis of citrus CcGASA gene family members. Syntenic regions and chromosomal regions are depicted in different colors. [file 12870_2021_3326_MOESM8_ESM.jpg]

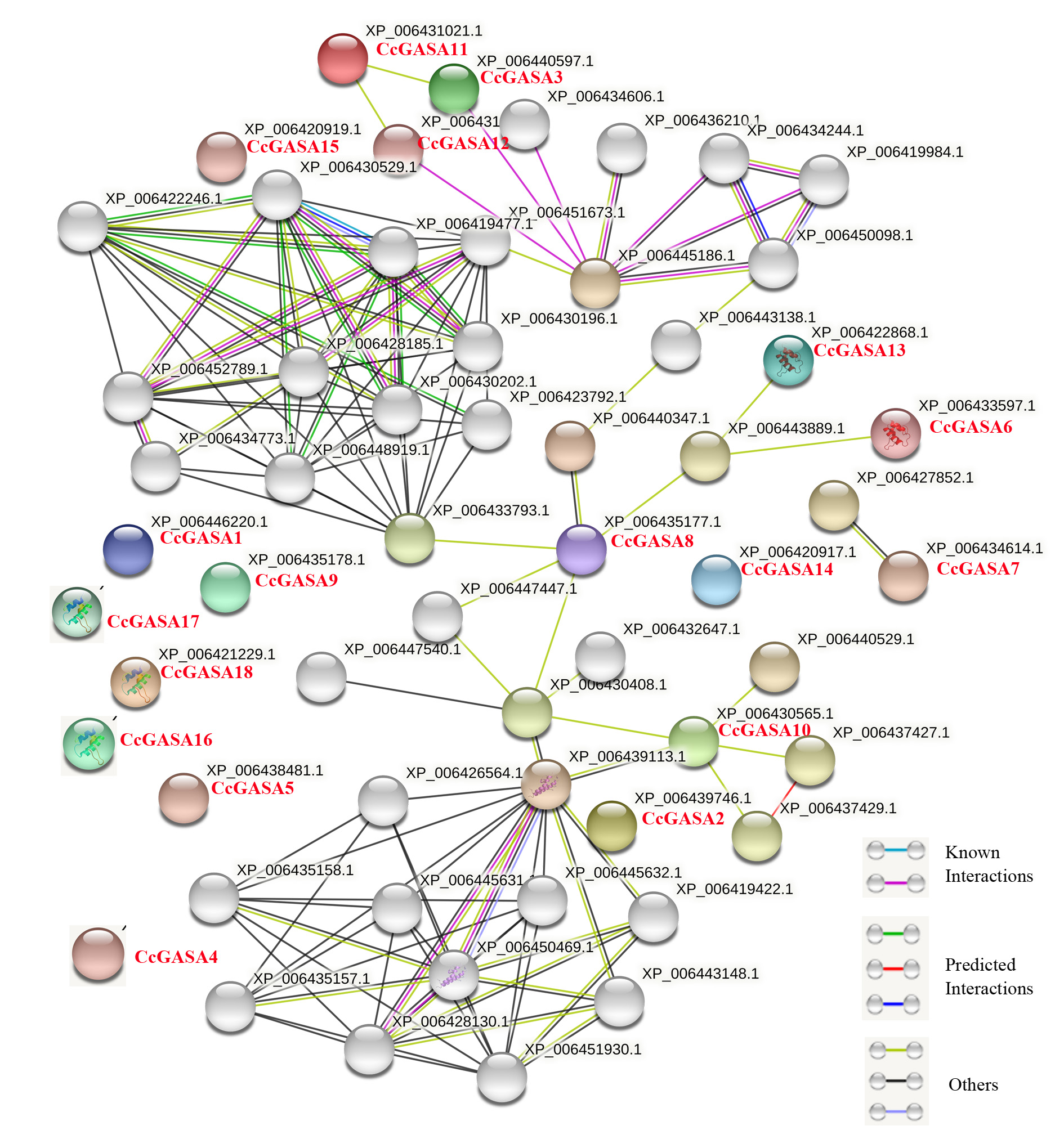

Supplement: Supplementary file 9 — Additional file 9: Figure S8. Putative protein-protein interaction network of CcGASA proteins in C.clementina. Colored nodes: query proteins and first shell of interactors. white nodes: second shell of interactors. Empty nodes: proteins of unknown 3D structure. Filled nodes: some 3D structure is known or predicted. [file 12870_2021_3326_MOESM9_ESM.jpg]
